# Heat But Not Mechanical Hypersensitivity Depends on Voltage-Gated Ca_V_2.2 Calcium Channel Activity in Peripheral Axon Terminals Innervating Skin

**DOI:** 10.1523/JNEUROSCI.0195-21.2021

**Published:** 2021-09-08

**Authors:** Daniel M. DuBreuil, Eduardo Javier Lopez Soto, Simon Daste, Remy Meir, Daniel Li, Brian Wainger, Alexander Fleischmann, Diane Lipscombe

**Affiliations:** ^1^Carney Institute for Brain Science and Department of Neuroscience, Brown University, Providence, Rhode Island 02912; ^2^Departments of Neurology and Anesthesia, Critical Care and Pain Medicine, Massachusetts General Hospital, Harvard Medical School, Boston, Massachusetts 02114

**Keywords:** cacna1b, calcium channel, conotoxin, hyperalgesia, N-type calcium channels, nociceptor

## Abstract

Voltage-gated Ca_V_2.2 calcium channels are expressed in nociceptors at presynaptic terminals, soma, and axons. Ca_V_2.2 channel inhibitors applied to the spinal cord relieve pain in humans and rodents, especially during pathologic pain, but a biological function of nociceptor Ca_V_2.2 channels in processing of nociception, outside presynaptic terminals in the spinal cord, is underappreciated. Here, we demonstrate that functional Ca_V_2.2 channels in peripheral axons innervating skin are required for capsaicin-induced heat hypersensitivity in male and female mice. We show that Ca_V_2.2 channels in TRPV1-nociceptor endings are activated by capsaicin-induced depolarization and contribute to increased intracellular calcium. Capsaicin induces hypersensitivity of both thermal nociceptors and mechanoreceptors, but only heat hypersensitivity depends on peripheral Ca_V_2.2 channel activity, and especially a cell-type-specific Ca_V_2.2 splice isoform. Ca_V_2.2 channels at peripheral nerve endings might be important therapeutic targets to mitigate certain forms of chronic pain.

**SIGNIFICANCE STATEMENT** It is generally assumed that nociceptor termini in the spinal cord dorsal horn are the functionally significant sites of Ca_V_2.2 channel in control of transmitter release and the transmission of sensory information from the periphery to central sites. We show that peripheral Ca_V_2.2 channels are essential for the classic heat hypersensitivity response to develop in skin following capsaicin exposure. This function of Ca_V_2.2 is highly selective for heat, but not mechanical hypersensitivity induced by capsaicin exposure, and is not a property of closely related Ca_V_2.1 channels. Our findings suggest that interrupting Ca_V_2.2-dependent calcium entry in skin might reduce heat hypersensitivity that develops after noxious heat exposure and may limit the degree of heat hypersensitivity associated with certain other forms of pain.

## Introduction

Voltage-gated calcium channels Ca_V_2.2 (N-type current) and Ca_V_2.1 (P-type current) are the primary sources of calcium that control neurotransmitter release from nociceptor presynaptic termini in the spinal cord dorsal horn (Diaz and Dickenson, 1997; Heinke et al., 2004; Motin and Adams, 2008; Jayamanne et al., 2013). Opioids act through µ-opioid receptors and inhibit the gating of presynaptic Ca_v_2.2 channels in the spinal cord dorsal horn and decrease synaptic transmission (Horváth et al., 2001; Andrade et al., 2010). Ca_V_2.2 channels in nociceptors are also targets of non-opioid analgesics including the selective Ca_V_2.2 channel inhibitor ω-conotoxin MVIIA (ω-CgTx MVIIA, Prialt, Ziconotide, or SNX-111; Miljanich and Ramachandran, 1995; Bowersox et al., 1997; Miljanich, 2004; Patel et al., 2018). Intrathecal ω-CgTx MVIIA mitigates otherwise intractable pain in humans, and it is effective in animal models of inflammatory and nerve-injury-induced pain while having less effect on acute pain responses (Miljanich and Ramachandran, 1995; Bowersox et al., 1997; Miljanich, 2004; Bannister et al., 2017). However, clinical use of Ca_V_2.2 channel inhibitors is limited because of side effects because of inhibition of Ca_V_2.2 channels in the brain and sympathetic nervous system (Miljanich, 2004). A Ca_V_2.2 channel inhibitor with greater selectivity against chronic pain would have significant value.

Ca_V_2.2 channels are expressed throughout nociceptors at presynaptic terminals, soma, and peripheral nerves, but their biological significance at sites beyond a well-accepted role at presynaptic terminals is not well understood. Nociceptor free nerve endings in skin release inflammatory signaling molecules, including neuropeptides calcitonin gene-related peptide (CGRP), ATP, and substance P, which contribute to inflammatory hypersensitivity to non-noxious and noxious stimuli (Louis et al., 1989; Costigan and Woolf, 2000; Cook and McCleskey, 2002; Scholz and Woolf, 2002; Costigan et al., 2009). The signaling molecules that control release of pro-inflammatory molecules are not well characterized, although TRPA1 receptors have been proposed to play a role in peptide release (Lin King et al., 2019). A role for Ca_V_ channels is often assumed, but direct evidence for their involvement is lacking (Chiu et al., 2012). Interestingly, White and Cousins (1998) showed >20 years ago that daily intradermal inhibition of Ca_V_2.2, but not Ca_V_2.1 channels attenuates mechanical hyperalgesia in a peripheral nerve injury model, but this finding has not been pursued. More recently, Lee and colleagues (Lee et al., 2019) showed that external application of an inhibitor of both Ca_V_2.2 and Na_V_1.7/1.8 channels (CNCB-2) attenuates thermal hyperalgesia and mechanical hyperalgesia/allodynia induced in postoperative and inflammatory pain models, but these studies did not directly address the contributions of Ca_V_2.2 separate from Na_V_ channels.

Ca_V_2.2 channel α1 subunits are encoded by the *Cacna1b* gene, which generates splice isoforms with different functional characteristics across tissue and cell type, developmental age, and disease state (Lipscombe, 2005; Lipscombe et al., 2013; Lipscombe and Lopez Soto, 2019). Trpv1-lineage nociceptors express a unique Ca_V_2.2 splice isoform, Ca_V_2.2-e37a, whereas most other neurons only express Ca_V_2.2-e37b (Bell et al., 2004; Altier et al., 2007; Jiang et al., 2013; Lopez Soto et al., 2019), and Ca_V_2.2-e37a channels are more sensitive to modulation by morphine compared with Ca_V_2.2-e37b (Bell et al., 2004; Castiglioni et al., 2006; Altier et al., 2007; Raingo et al., 2007; Andrade et al., 2010; Marangoudakis et al., 2012; Jiang et al., 2013; Macabuag and Dolphin, 2015). Ca_V_2.2-e37a channels appear to be particularly critical for some forms of hyperalgesia, although Ca_V_2.2 splice isoforms contribute equally to acute nociception (Altier et al., 2007; Andrade et al., 2010).

Here, we present functional evidence that Ca_V_2.2 channels in Trpv1-lineage nociceptors are at peripheral nerve endings in skin in addition to their accepted role at central synapses. At central Trpv1-lineage nociceptor termini, Ca_V_2.2 channels contribute to the accurate transmission of information about acute noxious stimuli. Most significantly, we show that in the periphery, Ca_V_2.2 channels, and in particular Ca_V_2.2-e37a isoforms, are necessary for capsaicin-induced heat hypersensitivity.

## Materials and Methods

All mice used in this work, except for mice used for in culture calcium imaging, were bred at Brown University, and all protocols and procedures were approved by the Brown University Institutional Animal Care and Use Committee. For *in vitro* calcium imaging, mice were bred at Massachusetts General Hospital, and all protocols and procedures were approved by the Massachusetts General Hospital Institutional Animal Care and Use Committee. For all behavioral experiments, both male and female mice were included. For physiology experiments, sex was not identified before tissue collection. All values shown are mean ± SE. All behavior experiments assessing heat and mechanical sensitivity were conducted under blinded conditions; however, Ca_V_2.2 KO mice are obviously hyperactive relative to WT mice, which made genotype blinding ineffective in behavioral experiments using Ca_V_2.2 KO mice.

### 

#### 

##### Mice

*Trpv1-Cre* [Cavanaugh et al., 2011; catalog #JAX:017769; RRID: IMSR_JAX:017769], *lox-STOP-lox-ChR2-EYFP* (hereafter *ChR2-EYFP*; Madisen et al., 2012; catalog #JAX:012569; RRID: IMSR_JAX:012569,), *lox-STOP-lox*-*tdTomato* (hereafter *tdTomato*; Madisen et al., 2010; IMSR, catalog #JAX:007908; RRID: IMSR_JAX:007908), and *lox-STOP-lox^GCaMP6f^* (catalog #JAX: 028865) mice were purchased from The Jackson Laboratory. *Trpv1* is expressed in testes during gamete production (Maccarrone et al., 2005; Mizrak and van Dissel-Emiliani, 2008; Francavilla et al., 2009) inducing Cre-dependent reporter expression in spermatozoa. We confirmed widespread reporter expression beyond the Trpv1-lineage in offspring of *Trpv1*^Chr2EYFP+/−^ or *Trpv1*^tdTomato+/−^ mice; therefore, all mice used in this study were first-generation progeny of single homozygous parents. *Trpv1*-Cre^+/+^ mice were mated with either *ChR2-EYFP*^+/+^, *tdTomato*^+/+^, or *GCaMP6f*^+/+^ mice to generate *Trpv1*^Chr2EYFP+/−^, *Trpv1*^tdTomato+/−^, and *Trpv1*^GCaMP6f+/−^ offspring.

**Figure 1.**
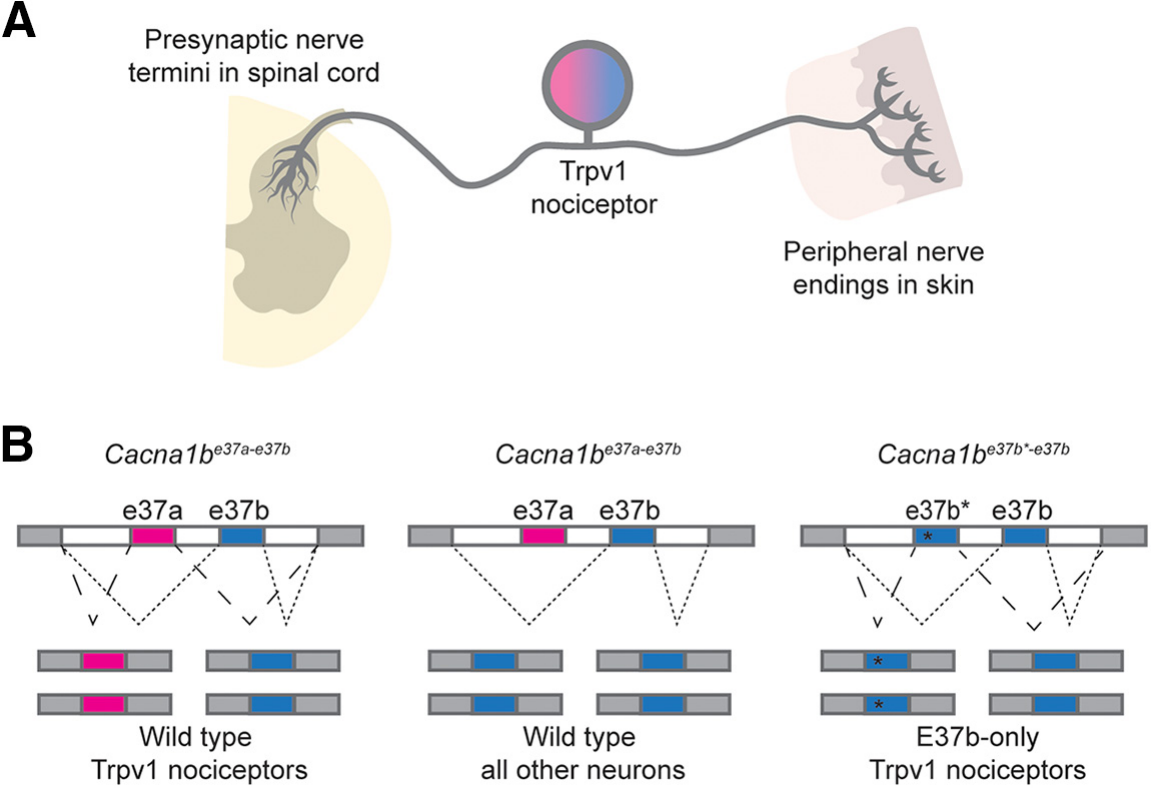
Trpv1-lineage nociceptors express Ca_V_2.2-e37a and Ca_V_2.2-e37b splice isoforms. ***A***, Trpv1-lineage nociceptor is depicted with presynaptic termini in spinal cord dorsal horn, soma, and nerve endings in skin. The magenta/blue color in the soma represents the two Cacna1b splice isoforms that dominate in these neurons. ***B***, Schematic of mutually exclusive pattern of alternative splicing for e37a and e37b in Trpv1-lineage nociceptors and all other neurons in wild-type (*Cacna1b^e37a-e37b^*) mice, as well as in Trpv1-lineage nociceptors of e37b-only *Cacna1b*^e37b*-*e37b*^ mice (Andrade et al., 2010).

Generation of *Cacna1b*^e37b^*^−37b+/+^ mice was described previously (Fig. 1*B*; Andrade et al., 2010). Wild-type *Cacna1b* express both e37a and e37b splice isoforms in Trpv1-lineage neurons (Bell et al., 2004; Andrade et al., 2010; López Soto and Lipscombe, 2020). We use our unique *Cacna1b*^e37b*-*e37b*+/+^ (e37b-only/e37b-e37b) mouse strain and compare with WT (*Cacna1b^e37a-e37b^*) to understand the unique action of e37a in Ca_V_2.2-dependent behavioral responses. Critically, we substituted e37a for e37b to generate *Cacna1b*^e37b*-*e37b*+/+^ (e37b-only) that lacks e37a but with WT protein levels (Fig. 1*B*). In Andrade et al., 2010 we found no evidence of up or down regulation of Ca_V_2.2 in e37b-e37b mice. We also found no compensatory alterations in expression of closely related Ca_V_2.1 channel proteins. Thus, any phenotypic difference between WT and e37b-only mice can be attributed to the loss of unique features of e37a. By contrast, when using exon-specific siRNAs, Ca_V_2.2 protein levels are significantly reduced (Altier et al., 2007). We generated a Ca_V_2.2 knock-out (KO) mouse strain by insertion of an EGFP+stop cassette in frame in exon 1 of the *Cacna1b* gene. All primers used in the creation of the Ca_V_2.2-KO mouse strain are shown in Table 1. To create the *Cacna1b*^−/−^ targeting construct, a 12 kb NsiI fragment from the 129S mouse BAC genomic clone bMQ122-B9 (Source BioScience) was cloned into the PstI site of pBSSK^+^. An AgeI site was inserted into exon 1 of *Cacna1b* by mutagenesis using a QuickChange II XL Site-Directed Mutagenesis Kit (StrataGene) with primers 1-For and 1-Rev. The EGFP+stop cassette was inserted in-frame into exon 1 at the AgeI site following amplification from pEGFP-C1 (BD Biosciences) using primers 2-For and 2-Rev. The loxP-NeoR-loxP cassette was inserted at the MluI site in the intron between exons 1 and 2 following PCR amplification from pL452 (Addgene) using primers 3-For and 3-Rev. The final targeting construct was 14.8 kb.

**Table 1.** Primers for generation of Ca_V_2.2-null mouse strain

Strain	Primer	Sequence
Ca_V_2.2-KO construct generation	1-For	GAACTGCTTCACCGTCAACCGGTCGCTCTTCGTCTTCAGC
	1-Rev	GCTGAAGACGAAGAGCGACCGGTTGACGGTGAAGCAGTTC
	2-For	CTAGACCGGTCAATGGTGAGCAAGGGCGAGGAGC
	2-Rev	CTAGACCGGTGACAAACCACAACTAGAATGC
	3-For	CATGACGCGTGAATTCCTGCAGCCCAATTCC
	3-Rev	CTAGACGCGTCCCTCGAGGGACCTAATAACTTCG
Ca_V_2.2-KO targeting	4-For-Left	GTGCATGTGTTTATCTGTGTG
	4-Rev-Left	GAACTTCAGGGTCAGCTTGC
	5-For-Right	CTACCCGGTAGAATTTCGACG
	5-Rev-Right	GGTCCTGCATGTAGGCCTCC

Mouse 129Ola ES cells derived from a male embryo were grown on mitotically inactive SNL76/7 feeder cells. Ten million (10^7^) ES cells were electroporated with 20 µg of a construct linearized with PvuI, and G418 selection was initiated after 24 h. Correctly targeted ES cell clones were identified by PCR and injected in E3.5 blastocysts isolated from C57Bl/6-*Tyrc-Brd* female mice. Injected blastocysts were implanted into day 2.5 pseudopregnant females for the generation of chimeras. Male chimeras were mated with C57BL/6-*Tyrc*-*Brd* females to obtain F1 progeny. Germ line transmission was confirmed by PCR of genomic DNA from each ES cell clone to confirm homologous integration from the long arm and short arm of the targeting vector using primers indicated in Table 1 for the left and right arms. The neomycin resistance cassette was subsequently removed from F1 mice by crossing to a B6.FVB-Tg(EIIa-cre)C5379Lmgd/J (The Jackson Laboratory; RRID: IMSR_JAX:003724). Deletion of the neomycin cassette was confirmed by PCR amplification of genomic DNA.

Individual WT strains were generated in parallel from the same genetic background used to create *Cacna1b*^−/−^ and *Cacna1b*^e37b*-*e37b*^ mouse strains. The two WT strains were pooled together following determination that there were no significant differences on any assessments.

##### Acute nociception assays

Responses to heat, mechanical, and LED stimulation were analyzed from male and female *Cacna1b*^+/+^/*Trpv1*^Chr2EYFP+/−^, *Cacna1b*^−/−^/*Trpv1*^Chr2EYFP+/−^, and *Cacna1b*^e37b*-e37b+/+^/*Trpv1*^Chr2EYFP+/−^ mice. Mice were between 2 and 4 months old. In total, 195 *Cacna1b*^+/+^/*Trpv1*^Chr2EYFP+/−^ mice, 89 *Cacna1b*^−/−^/*Trpv1*^Chr2EYFP+/−^, and 75 *Cacna1b*^e37b*-e37b+/+^/*Trpv1*^Chr2EYFP+/−^ mice were used to monitor the following acute nocifensive responses: heat (146 WT, 56 Ca_V_2.2-null, 42 Ca_V_2.2-e37b), mechanical (32 WT, 24 Ca_V_2.2-null, 26 Ca_V_2.2-e37b), and LED (17 WT, 9 Ca_V_2.2-null, 7 Ca_V_2.2-e37b).

We used a Plantar Analgesia Meter (IITC Life Science) to assess heat responses to radiant heat. Mice were placed in Plexiglas containers on an elevated glass plate and allowed to habituate for 1 h before testing. A visible-light, radiant heat source was positioned beneath the mice and aimed using low-intensity visible light to the plantar surface of the hindpaw. An orange-pass filter was used to prevent blue-light activation of channelrhodopsin in sensory nerve terminals. Trials began once the high-intensity light source was activated and ended once the mouse withdrew a hindpaw and (1) shook the paw, (2) licked the paw, or (3) continued to withdraw the paw from stimulation. Immediately on meeting response criteria, the high-intensity light source was turned off. The latency to response was measured to the nearest 0.01 s for each trial using the built-in timer, which is activated and deactivated with the high-intensity beam. For all trials, the high-intensity beam was set at 40%, the low-intensity beam set at 10%, and the maximum trial duration was 30 s. Three trials were conducted on each hindpaw for each mouse, with at least 1 min between trials of the same hindpaw (Hargreaves et al., 1988).

To assess responses to mechanical stimuli, we used a Dynamic Plantar Aesthesiometer (catalog #37450, Ugo Basile). Mice were placed in a Plexiglas container over an elevated mesh platform for at least 30 min to allow accommodation before the measurement. The plantar surface of the hindpaw was stimulated with a 0.5 mm filament pushed against the plantar side of the hindpaw with linear ascending force, from 0–5 × *g* over 10 s in 0.5 × *g*/s intervals, until a fast withdraw of the paw was observed or a maximum trial time of 20 s elapsed. The latency to response in seconds and actual force to the nearest 0.1 × *g* at the time of paw withdrawal were automatically detected and recorded by the unit. Three trials were conducted on each hindpaw for each mouse with at least 1 min between trials of the same hindpaw.

For assessing nocifensive responses to optogenetic nociceptor activation, male or female mice were placed in Plexiglas containers on an elevated glass surface. A fiber-coupled, blue (465 nm) LED light (Plexon) was mounted to a movable stage at a fixed distance below the glass platform. Light intensity was controlled by the supplied driver, and intensity (1–9 mW, ∼0.1–1 mW/mm^2^) was measured using a light meter (PM100A S121C, Thorlabs) mounted on top of the glass plate. Mice were allowed to habituate to the chamber for 1 h before testing. Each mouse was stimulated 10 times, equally divided between the left and right hindpaw, at each of six light intensity levels for a total of 60 trials per mouse. In each trial, the LED light was directed at the plantar surface of one hindpaw for 5 s or until a nocifensive response was elicited. Nocifensive responses included hindpaw withdrawal accompanied by at least one of the following: shaking or licking the stimulated hindpaw or continued hindpaw withdrawal during prolonged stimulation. All responses occurred within 3 s of stimulus onset. The number of nocifensive trials was counted for each mouse at each light intensity level, and response distributions for individual mice were fit using a four-parameter logistic curve as follows:

$$y=min-\frac{\left(max-min\right)}{1+{\left(\frac{x}{EC50}\right)}^{Hillslope}}.$$

Values for minimum, maximum, EC_50_, and Hill slope were calculated automatically, and mean values were used to construct a fit of the average response distribution.

All data acquisition and analyses were conducted under blinded conditions. Results were similar in unblinded experiments conducted independently by three different experimenters.

##### Immunohistochemistry

*Cacna1b*^+/+^/*Trpv1*^Chr2EYFP+/−^ and *Cacna1b*^+/+^/*Trpv1*^tdTomato+/−^ male mice (2–6 months) were transcardially perfused with cold PBS, followed by 4% paraformaldehyde (PFA) in PBS (phosphate-buffered saline). Spinal cord (L4–L6) and skin (hindpaw) were removed and postfixed in 4% PFA overnight at 4°C. Samples were cryoprotected in 30% sucrose in PBS with Triton X-100 at 4°C for 48 h, frozen in optimal cutting temperature (OCT) solution and cut into 14 μm slices.

Slices were blocked overnight using 5% bovine serum albumin (BSA; Sigma-Aldrich) in PBS with 0.4% Triton X-100 (PBST; Sigma). Anti-CGRP (catalog #PC205L, Millipore; RRID: AB_2068524) antibody was applied in PBST with 5% BSA at 1:250 dilution for 48 h at 4°C. Secondary antibodies Alexa Fluor 488 donkey-anti-rabbit (RRID: AB_2571722) and Alexa Fluor 488 donkey anti-mouse (RRID: AB_2571721) were applied at 1:200 in PBST with Alexa Fluor 647 conjugated Isolectin B4 (catalog #SCR_014365, Thermo Fisher) at 1:100 and DAPI (catalog #62248, Thermo Fisher) at 1:1000 for 4 h at room temperature. Images were collected using a Zeiss LSM 800 confocal microscope using ZEN software.

##### RNAScope in situ hybridization

RNAScope (Advanced Cell Diagnostics) *in situ* hybridization was performed as instructed in the manual, with the following differences. DRGs were isolated from *Cacna1b*^+/+^, *Cacna1b*^−/−^, and *Cacna1b* ^e37b*-e37b+/+^ mice and processed and sectioned as described (see above, Immunohistochemistry). Following sectioning, slices were dehydrated with 100% ethanol for 5 min and allowed to air dry. A barrier was drawn around each section using a hydrophobic barrier pen. Slides were incubated at 40°C in Protease III for 30 min. *Cacna1b* or *Cacna1b*-e37a probes were applied at 40°C for 4 h and detected using amplification reagents 1–4, as described in the kit. Images were collected using a Zeiss LSM 800 confocal microscope using ZEN software. At least three slides from two mice (2 months old) were used for analysis. Probe specificity was confirmed using Ca_V_2.2-null and Ca_V_2.2-e37b samples.

##### Spinal cord slice physiology

Acute spinal cord slices were prepared from *Cacna1b*^+/+^/*Trpv1*^Chr2EYFP+/−^, *Cacna1b*^−/−^/*Trpv1*^Chr2EYFP+/−^, and *Cacna1b*^e37b*-e37b+/+^/*Trpv1*^Chr2EYFP+/−^ mice [mixed gender, postnatal day (P)14–21]. Mice were anesthetized by intraperitoneal injection of Beuthanasia-S and transcardially perfused with cold oxygenated artificial CSF (aCSF) containing the following (in mm): 125 NaCl, 2.5 KCl, 26 NaHCO_3_, 1.25 NaH_2_PO_4_, 1.5 CaCl_2_, 6 MgCl_2_, 25 Glucose, and 1 Kynurenic acid. The spinal cord was removed, embedded in 2% low-melting agarose, and cut into 300 μm sections using a Leica VT1200S vibrating blade microtome in the same aCSF solution. Sections were then transferred to a holding chamber containing the same aCSF solution at 30°C for 1 h and at room temperature thereafter. For recording, individual slices were transferred to a recording chamber and continually perfused with oxygenated aCSF containing the following (in mm): 119 NaCl, 2.5 KCl, 26 NaHCO_3_, 1 NaH_2_PO_4_, 2.5 CaCl_2_, 1.3 MgSO_4_, 25 Glucose, and 1.3 Na-Ascorbate. Patch pipettes were filled with an internal solution containing the following (in mm): 125 KGluconate, 28 NaCl, 2 MgCl2, 2 Mg-ATP, 0.3 Na-GTP, 0.6 EGTA, and 10 HEPES and had a resistance between 3 and 5 MΩ. Synaptic responses were elicited by 1 ms pulses of blue light through the 40× microscope objective controlled by a shutter, and the blue light was focused directly on the recorded cell. Cells were held at −70 mV during all protocols.

##### Inflammatory models

*Cacna1b*^+/+^/*Trpv1*^Chr2EYFP+/−^, *Cacna1b*^−/−^/*Trpv1*^Chr2EYFP+/−^, and *Cacna1b*^e37b*-e37b+/+^/*Trpv1*^Chr2EYFP+/−^ mice (male and female, 4–6 months) were anesthetized with isoflurane. Capsaicin (0.1% w/v, in sterile saline with 5% Tween 20, 20 µL), A438079 (6 mm in sterile saline, 20 µL), ω-conotoxin MVIIA (1 μm in sterile saline, 20 µL), or ω-Agatoxin IVA (1 μm in sterile saline, 20 µL) was injected via a 30-gauge needle into the left hindpaw, and mice were allowed to recover in the testing chamber. Heat or mechanical sensitivity was assessed using the radiant heat or automated von Frey assays, respectively (described above). Median response latency of at least three trials at each time point was used to assess sensitivity. To compare effect size among different mouse strains we applied the following formula: [(Effect(Mouse A) − Effect(WT))/Effect(WT)] × 100%.

##### Interleukin-1 analysis

WT and KO mice (*n* = 3 mice per genotype) were anesthetized with isoflurane and killed via intraperitoneal injection of euthanasia solution. The plantar skin from the left and right hindpaws was removed and ∼20 mg of skin was placed in 1 ml DMEM supplemented with 10% heat-inactivated fetal bovine serum and Penicillin-Streptomycin (Invitrogen). Skin cultures were maintained at 37°C with 5% CO_2_ for 72 h. One day before collection of conditioned media, HEK-Blue IL-1 cells (InvivoGen) were split into a 24-well plate with 0.5 ml DMEM supplemented with 10% heat-inactivated fetal bovine serum, Hygromycin, and Zeocin. Conditioned media containing IL-1 was collected from skin cultures, and 200 μl was added to each well of HEK-Blue cells for 24 h. Secreted embryonic alkaline phosphatase was detected by mixing 200 μl Quanti-Blue media with 50 μl HEK-Blue cell media in triplicate in a 96-well plate and quantified by reading the absorbance at 620 nm using a Biotek Synergy HTX Multi-Mode Microplate Reader at 37°C. Quanti-Blue media alone was used as a blank sample. This assay does not distinguish between IL-1α and IL-1β.

##### Calcium imaging in dissociated DRG

Dissociated nociceptors were prepared from C57BL/6J mice (Charles River Laboratories; unidentified gender, P14–28, *n* = 3 mice). Ganglia (∼40) were removed postmortem from the rostral-caudal extent of the vertebral column, collected, and placed into DMEM/F12 with 10% FBS. Cells were dissociated for 60 min using collagenase A (2 mg/ml; catalog #10103578001, Sigma) and dispase (2 mg/ml; catalog #17105041, ThermoFisher) followed by trituration with a fire-polished Pasteur pipette. Cells were filtered using a 70 µm filter followed by a 10% BSA gradient. Cells were resuspended in Neurobasal media supplemented with B27, Glutamax, Pen/Strep, and Nerve Growth Factor (256-GF, 0.1 mg/ml; R&D Systems) and plated into 96-well plates (catalog #89626, Ibidi) precoated with 2 µL dots of poly-D-lysine (0.1 mg/ml; catalog #P6407, Sigma) and laminin (2.5 mg/ml; catalog #23017015, ThermoFisher). Dissociated neurons were maintained at 37°C with 5% CO_2_ in O_2_ for 4 d. On the day of imaging, cells were rinsed with bath solution containing the following (in mm): 140 NaCl, 5 KCl, 2 CaCl_2_, 1 MgCl_2_, 10 Glucose, and 10 HEPES, pH 7.3, with NaOH. Cells were then incubated in neurobasal media containing 3 mg/ml Fluo-4AM (catalog #F14201, ThermoFisher) for 30 min at room temperature. After 30 min, Fluo-4-containing media was replaced by an external solution. Fluo-4 signal was monitored using a MetaXpress Micro High Content Imager (Molecular Devices) at a frequency of 1 Hz. Stimuli (20 µL per stimulus) were automatically applied at 20 s intervals beginning 5 s after the first image acquired. Neuronal somata and neurites were identified using a custom analysis script in ImageJ (version 1.52p), and fluctuations in Fluo-4 intensity over time were analyzed using R (version 3.5.0).

##### Two-Photon functional imaging in anesthetized mice

We used 4–5 month male and female first-generation double heterozygous offspring from single homozygous *Trpv1^Cre^* (catalog #JAX:017769, The Jackson Laboratory) and *lox-STOP-lox^GCaMP6f^* (catalog #JAX:028865, The Jackson Laboratory) parents for two-photon imaging of Trpv1-nociceptors expressing GCaMP6f. Mice were initially anesthetized with 3% isoflurane and maintained under 2% isoflurane anesthesia on a temperature-controlled pad throughout the 35–45 min imaging session. Intravenous tail injections (100 µl) of Texas Red (70% v/v in saline) were used to visualize blood vessels. To reduce respiration-induced movement artifacts, the hindpaw was attached to the base with dental gum. Imaging was performed with a two-photon microscope (Ultima Investigator, Bruker) with a 16×, 0.8 numerical aperture, water-immersion objective (Nikon) at 1× zoom, 512 × 512 pixels for an 820 × 820 μm field of view. A dual input femtosecond pulse source laser (Chameleon Discovery, Coherent) was tuned to 920 nm. Images were taken at 5, 15, and 30 min following capsaicin (10 μl, 0.1% w/v in sterile saline) injection alone or co-injection with conotoxin (10 μl, 0.1% w/v in sterile saline), then z-stacks were acquired as follows. We started at the skin epithelial cell layer and imaged 200 μm deep using a step size of 3 μm (∼67 optical sections/stack). Each optical section is the average of 16 frames acquired at 30 Hz and a dwell time of 0.4 μs. To quantify the relative change of GCaMP6f fluorescence intensity, we constructed maximum projections of each image in a sub-z-stack using FIJI (https://imagej.net/Fiji). Individual nerves were identified visually after acquisition based on the presence of a positive GCaMP6f signal within 15 min. GCaMP6f signal regions of interest (ROIs) were defined manually at either a 5 or 15 min time point following capsaicin exposure. GCaMP6f ROIs were user defined using an optimal path method for the subset of optical sections containing a positive signal. We used this single time point to then manually track corresponding coordinates for the other two time points. The blood vessel network was used to localize ROI coordinates across time points. Our analysis shows that the capsaicin-induced GCaMP6f signal in Trpv1-nociceptors is reduced by ω-CgTx MVIIA, and because we do not include nonresponder termini, it is likely that this analysis underestimates the effect of ω-CgTx MVIIA. All data acquisition and analyses were conducted under blinded conditions.

##### Statistical analyses

**Figure 2.**
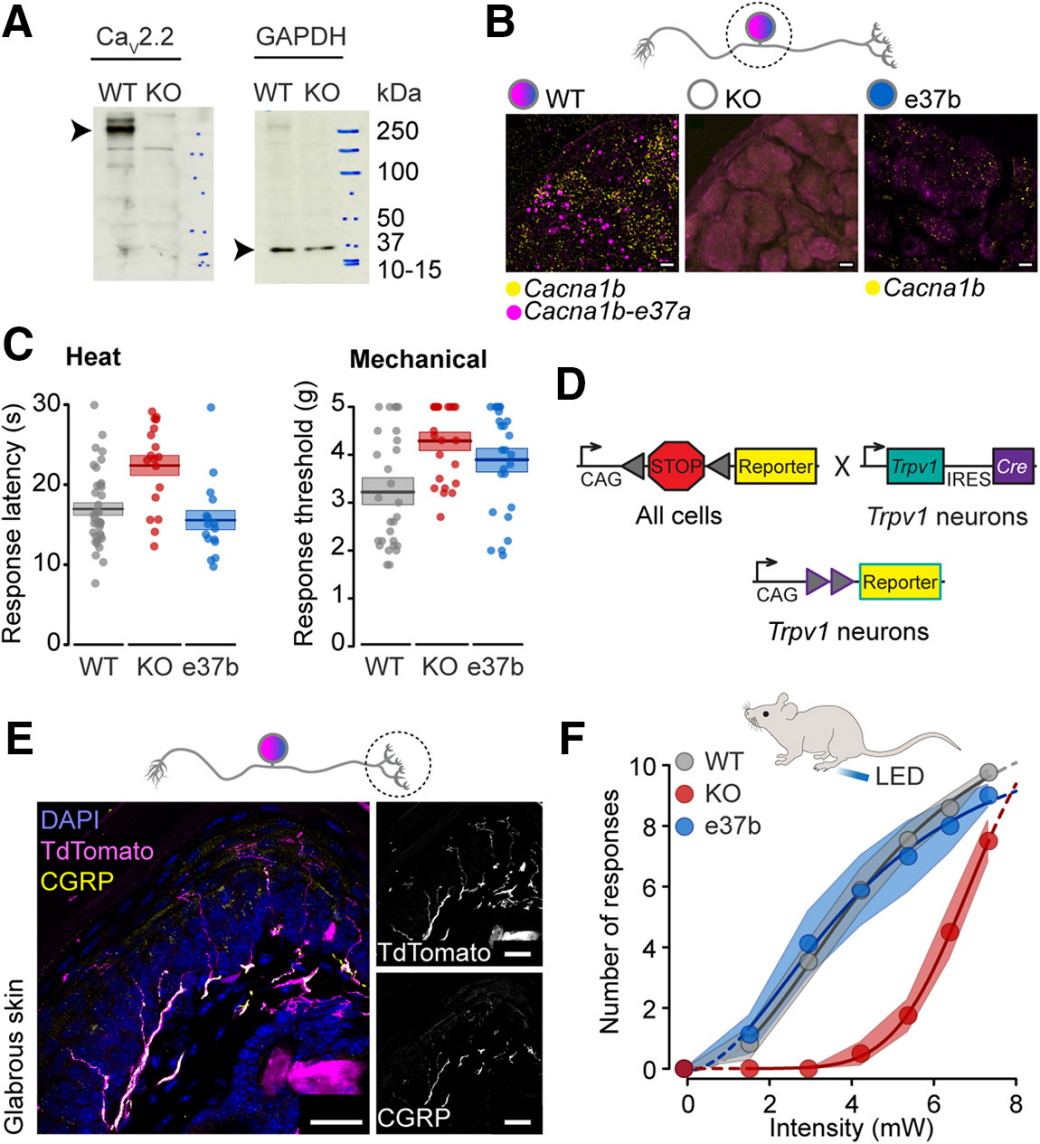
Ca_V_2.2 knock-out, but not Ca_V_2.2-e37b only, mice exhibit impaired heat and mechanical nociception compared with wild-type controls. ***A***, Left, Western blot shows major full-length Ca_V_2.2 α1 subunit protein (∼250 kDa band) in whole brain lysate from WT mice and its absence in Ca_V_2.2-null mice (KO). Additional minor lower molecular weight bands present in WT but not in KO may reflect different isoforms or proteolytic fragments (100–150 kDa) of Ca_V_2.2 α1. Additional minor bands present in both WT and KO brain lysates (∼150 kDa) reflect nonspecific binding of anti-Ca_V_2.2 antibody. Right, GAPDH signals (∼37 kDa) are present in WT and KO brain lysate in the same blot, stripped and reprobed with anti-GAPDH. ***B***, Visualization of Cacna1b mRNAs in DRG. Cacna1b mRNAs visualized with a pan probe (yellow) in WT mice, below detection in Ca_V_2.2 KO mice, and visible in *Cacna1b*^e37b*-*e37b*^-only mice (e37b). Cacna1b-37a mRNA (magenta) signals visible in WT, and fewer, scattered signals in e37b-only mice. Scale bars: 10 μm. ***C***, Heat and mechanical sensitivity of Ca_V_2.2 WT (gray), KO (red), and Ca_V_2.2-e37b-only (blue) mice. Heat and mechanical sensitivities of KO mice are reduced compared with WT and e37b-only mice. Each value shown represents the median of three measurements from the following number of mice (heat, WT *n* = 38, KO *n* = 18, e37b *n* = 16; mechanical: WT *n* = 32, KO *n* = 24, e37b *n* = 26), together with means (horizontal line) and SEs (shaded area). Mean ± SE was for heat latency; WT: 16.9 ± 0.8 s, KO: 22.4 ± 1.3 s; e37b: 15.5 ± 1.2 s; mechanical threshold WT: 3.35 ± 0.21 × g; KO: 4.29 ± 0.16 × g; e37b: 3.90 ± 0.21 × g; *t* and *p* values calculated by univariate ANOVA with Tukey's HSD correction for multiple comparisons are for heat WT versus KO, *t* = 3.845, *p* = 0.0008; WT versus e37b, *t* = −0.929, *p* = 0.624. Mechanical WT versus KO, *t* = 2.806, *p* = 0.021; WT versus e37b, *t* = 1.676, *p* = 0.228. ***D***, Scheme shows Cre/loxP breeding strategy to generate heterozygote reporter strains expressing either ChR2 or tdTomato (reporter) in Trpv1-lineage neurons. Trpv1 is expressed in testes during gamete production, so only first-generation progeny were used (see above, Materials and Methods). ***E***, Free nerve endings of Trpv1-lineage nociceptors in plantar glabrous skin coexpress CGRP. Left, Overlay shows Trpv1/tdTomato (magenta) free nerve endings, anti-CGRP (yellow), and cell nuclei labeled with DAPI (blue). Right, Grayscale images of *Trpv1/tdTomato* (top) and anti-CGRP (bottom) signals. Scale bars: 25 μm. ***F***, Behavioral responses induced by 5 s LED directed to plantar hindpaws of WT, KO, and e37b-only mouse strains expressing ChR2 in Trpv1-lineage neurons. KO mice were less responsive to 465 nm LED stimulation compared with WT and e37b mouse strains. Each symbol represents the mean ± SE of the number of responses to 10 separate LED stimuli, applied to the following number of mice: WT/Trpv1/ChR2-EYFP, *n* = 17; KO/Trpv1/ChR2-EYFP, *n* = 9; and e37b/Trpv1/ChR2-EYFP, *n* = 7. Intensity-response curves for each mouse were fit using a four-parameter logistic function; *t* and *p* values for ANOVA with Tukey's HSD correction for multiple comparisons are for WT versus KO: *t* = −2.607, *p* = 0.000002; and WT versus e37b: *t* = −0.880, *p* = 0.997.

**Figure 3.**
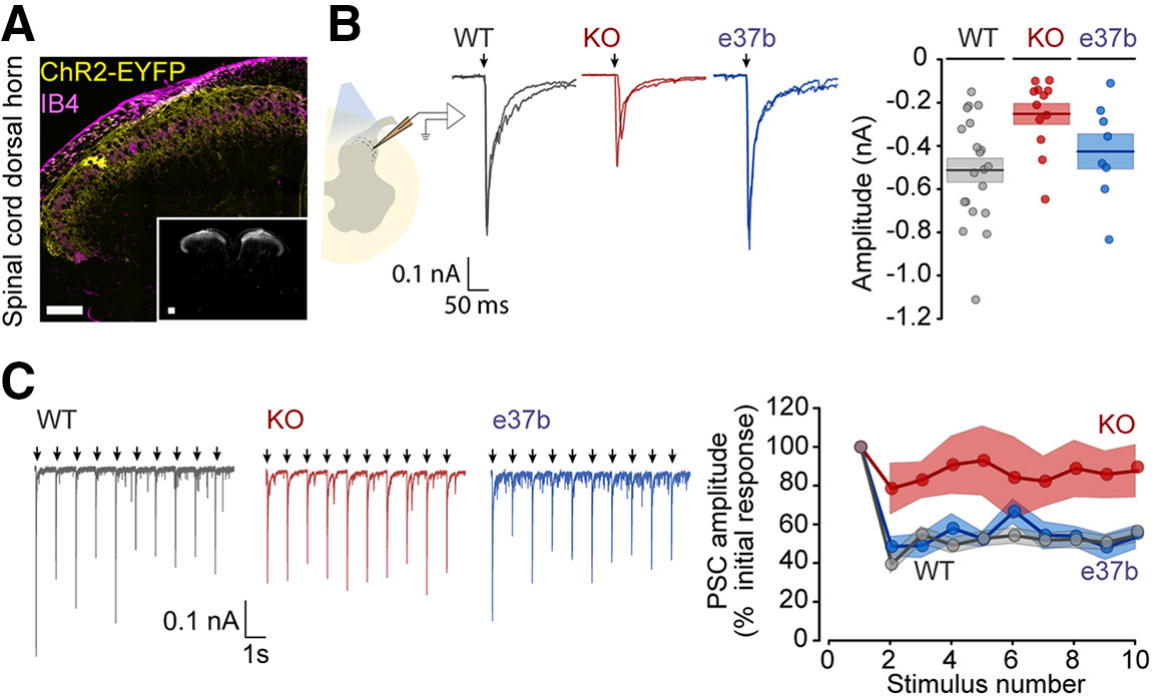
Synaptic currents in acute spinal cord slices from Ca_V_2.2 knockout, but not Ca_V_2.2-e37b-only, mice are reduced and show less paired pulse facilitation relative to wild-type controls. ***A***, Transverse section of lumbar spinal cord dorsal horn from *Cacna1b*^+/+^/*Trpv1*^Chr2EYFP+/−^ mouse showing ChR2-EYFP (yellow) and IB4 (magenta) to identify lamina II. Inset, Grayscale image of whole spinal cord section from *Cacna1b*^+/+^/*Trpv1*^Chr2EYFP+/−^ mouse showing reporter expression is restricted to dorsal horns. Scale bars: 100 μm. ***B***, Left, Scheme of experimental setup for recording light-activated synaptic currents in postsynaptic lamina II neurons. Middle, Synaptic currents in lamina II dorsal horn neurons of *Cacna1b*^+/+^/*Trpv1*^Chr2EYFP+/−^ (WT, gray), *Cacna1b*^−/−^/*Trpv1*^Chr2EYFP+/−^ (KO, red), and *Cacna1b*^37b*-37b+/+^/*Trpv1*^Chr2EYFP+/−^ (e37b, blue) mice. Right, Peak current amplitudes, symbols represent values of individual neurons, together with mean (horizontal line) ± SE (shaded area). Analyses of synaptic currents recorded from 20 WT, 12 KO, and 6–8 e37b neurons were as follows: mean ± SE and *t* and *p* values assessed by multivariate ANOVA were for peak amplitude, WT: −547.6 ± 76.0 pA, KO: −252.1 ± 48.2 pA, *t* = 3.202, *p* = 0.008 (WT vs KO), e37b: −425.3 ± 80.7 pA, *t* = 0.931, *p* = 0.950 (WT vs e37b); total charge, WT: −20.6 ± 2.5 pC, KO: −7.2 ± 1.1 pC, *t* = 4.975, *p* = 0.000051 (WT vs KO); e37b: −14.3 ± 2.5 pC, *t* = 1.448, *p* = 0.328 (WT vs e37b); weighted time constant, WT: 44.5 ± 5.9 ms, KO: 27.4 ± 2.3 ms, *t* = −2.749, *p* = 0.009 (WT vs KO), e37b: 32.7 ± 5.4 ms, *t* = −1.389, *p* = 0.431 (WT vs e37b); slow time constant, WT: 123.6 ± 12.9 ms, KO: 93.1 ± 8.4 ms, *t* = −2.103, *p* = 0.122 (WT vs KO), e37b: 107.6 ± 4.0 ms, *t* = −0.722, *p* = 0.577 (WT vs e37b); fast time constant, WT: 11.7 ± 1.6 ms, KO: 10.8 ± 0.9 ms, *t* = −1.167, *p* = 0.351 (WT vs KO), e37b: 13.8 ± 3.3 ms, *t* = 0.317, *p* = 0.995 (WT vs e37b); contribution of slow component to response amplitude, WT: 29.9 ± 3.0%, KO 22.0 ± 2.8%, *t* = −1.546, *p* = 0.282 (WT vs KO), e37b: 19.9 ± 4.8%, *t* = −1.626, *p* = 0.922 (WT vs e37b). The weighted time constant is a value that reflects the contribution of both fast and slow components in a given probability density function (fast, slow); it is the sum of the product of the amplitude and time constant for each component. Recordings from two neurons from e37b-only mice were better fit by single exponentials than double and were thus removed from kinetic analyses. ***C***, Left, Synaptic currents elicited by a series of 10 optical stimuli applied at 1 Hz from *Cacna1b*^+/+^/*Trpv1*^Chr2EYFP+/−^ (WT, *n* = 17, gray), *Cacna1b*^−/−^/*Trpv1*^Chr2EYFP+/−^ (KO, *n* = 7, red), and *Cacna1b*^37b-37b+/+^/*Trpv1*^Chr2EYFP+/−^ (e37b, *n* = 8, blue) mice. Right, Relative amplitude of postsynaptic currents compared with the initial response, evoked by optical stimulation at 1 Hz for WT (gray, *n* = 9 slices), KO (red, *n* = 7 slices), and e37b-only (blue, *n* = 8 slices) mice; *t* and *p* values assessed by repeated measures ANOVA and Tukey's HSD correction for multiple comparisons are for WT versus KO, *t* = 3.211, *p* = 0.001; WT versus e37b, *t* = −0.103, *p* = 0.940.

**Figure 4.**
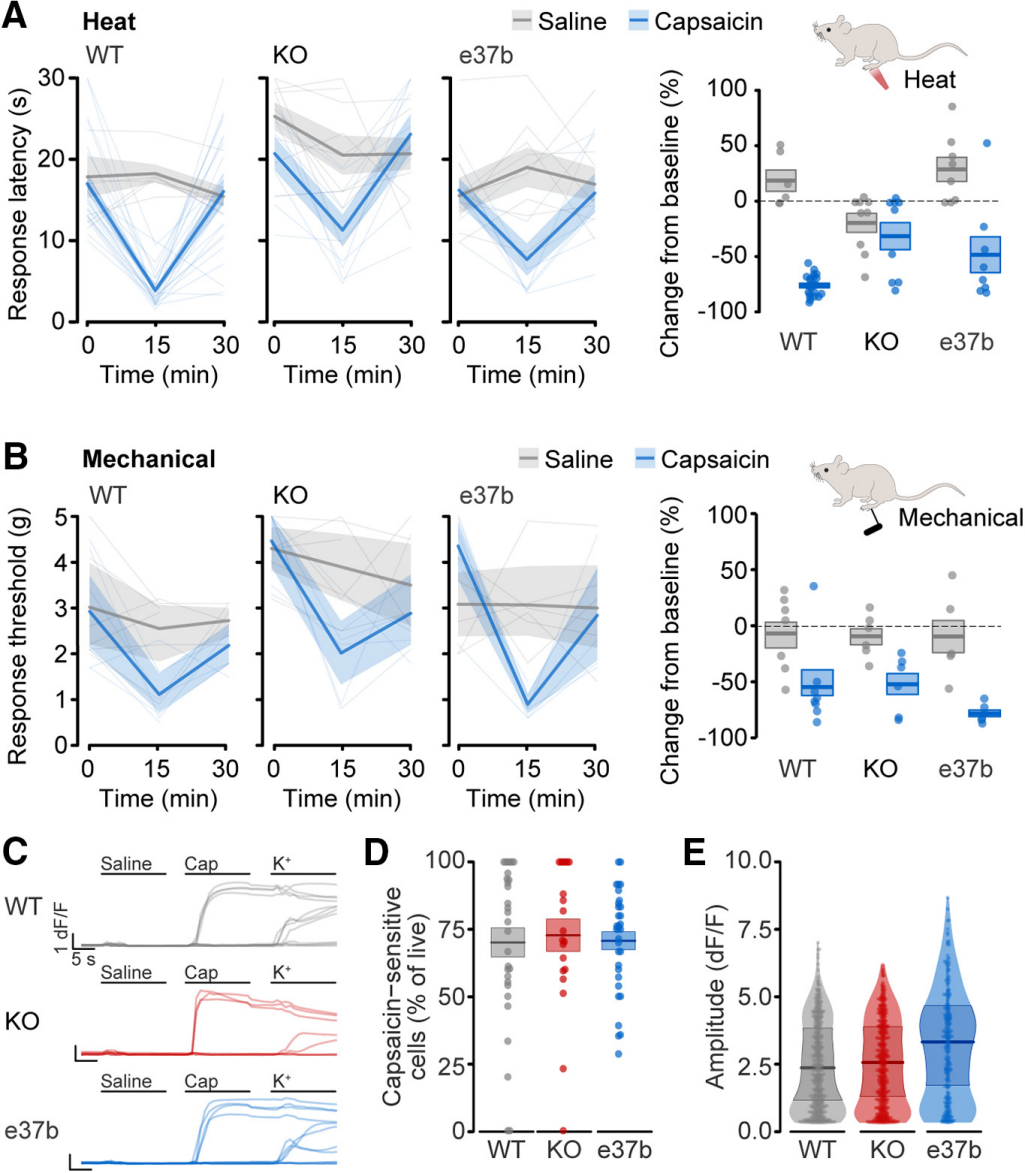
Capsaicin-induced heat hypersensitivity requires Ca_V_2.2 channels. ***A***, Heat sensitivity of plantar hindpaw of WT, KO, and e37b mice measured before (0 min), and 15 and 30 min after injection of 0.1% w/v capsaicin (blue) or saline (gray). Left, Responses from individual mice (thin lines, dashed and solid), average data (thick lines), and SE (shaded areas) are shown for all conditions. Right, Heat responses represented as change from baseline for individual mice (symbols, solid and open), average (horizontal line), and SE (shaded areas). Mean and SE are for WT/Saline: *n* = 6 mice, 19.7 ± 9.8%; WT/Capsaicin: *n* = 23 mice, −76.1 ± 2%; KO/Saline: *n* = 9 mice, −18.9 ± 8.8%; KO/Capsaicin: *n* = 9 mice, −31.1 ± 12.4%; e37b/Saline: *n* = 8 mice, 40.1 ± 12.1%; e37b/Capsaicin: *n* = 8 mice, 30.0 ± 11.0%; *t* and *p* values for the difference between saline and capsaicin effects calculated by ANOVA, and Tukey's HSD correction for multiple comparisons are for WT/Capsaicin versus KO/Capsaicin, *t* = 5.395, *p* = 0.000004; WT/Capsaicin versus e37b/Capsaicin, *t* = 2.751, *p* = 0.021. ***B***, Mechanical sensitivity of plantar hindpaw of WT, KO, and e37b mice measured before (0 min) and 15 and 30 min after injection of 0.1% w/v capsaicin (blue) or saline (gray). All experiments were performed under blinded conditions. Left, responses from individual mice (thin lines), average data (thick lines), and SE (shaded areas) are shown for all conditions. Right, Mechanical responses represented as change from baseline for individual mice (symbols), average (horizontal line), and SE (shaded areas). Mean and SE are for WT/Saline: *n* = 7 mice, −6.9 ± 12.8%; WT/Capsaicin: *n* = 8 mice, −54.7 ± 13.4%; KO/Saline: *n* = 6 mice, −9.4 ± 7.8%; KO/Capsaicin: *n* = 6 mice, −52.2 ± 10.5%; e37b/Saline: *n* = 6 mice, 16.1 ± 29.6%; e37b/Capsaicin: *n* = 6 mice, −78.3 ± 3.4%; *t* and *p* values calculated by univariate ANOVA and Tukey's HSD correction for multiple comparisons are for WT/Capsaicin versus KO/Capsaicin, *t* = −0.117, *p* = 0.994; WT/Capsaicin versus e37b/Capsaicin, *t* = 1.080, *p* = 0.996. ***C***, Example calcium responses to saline, capsaicin (200 nm) and high K^+^ (35 mm) in individual DRG neurons from Ca_V_2.2 WT (gray), KO (red), and Ca_V_2.2-e37b only (blue) mice. ***D***, Percentage of capsaicin-sensitive cells among live cells. Mean (line) ± SE (shaded area) are for WT: 70.1 ± 5.2%, *n* = 32 wells; KO: 72.8 ± 6.0%, *n* = 20 wells; e37b: 70.7 ± 3.4%, *n* = 32 wells; *t* and *p* values calculated by univariate ANOVA and Tukey's HSD correction for multiple comparisons are for WT v KO, *t* = 0.368, *p* = 0.928; WT versus e37b, *t* = 0.096, *p* = 0.995. ***E***, Peak amplitude of calcium responses to capsaicin among capsaicin-sensitive nociceptors. Plot shows distribution of response amplitudes with median (line) and interquartile range (dark shaded area), as well as individual response amplitudes. Median (interquartile range) amplitudes were for WT: 2.08 [2.81] df/*F*, *n* = 510 cells; KO: 2.31 [2.87] df/*F*, *n* = 474 cells; e37b: 3.14 [3.25] df/*F*, *n* = 283 cells. *P* values calculated by pairwise Wilcoxon test with Holm correction for multiple comparisons were for WT versus KO, *p* = 0.228 and WT versus e37b, *p* = 0.000005.

**Figure 5.**
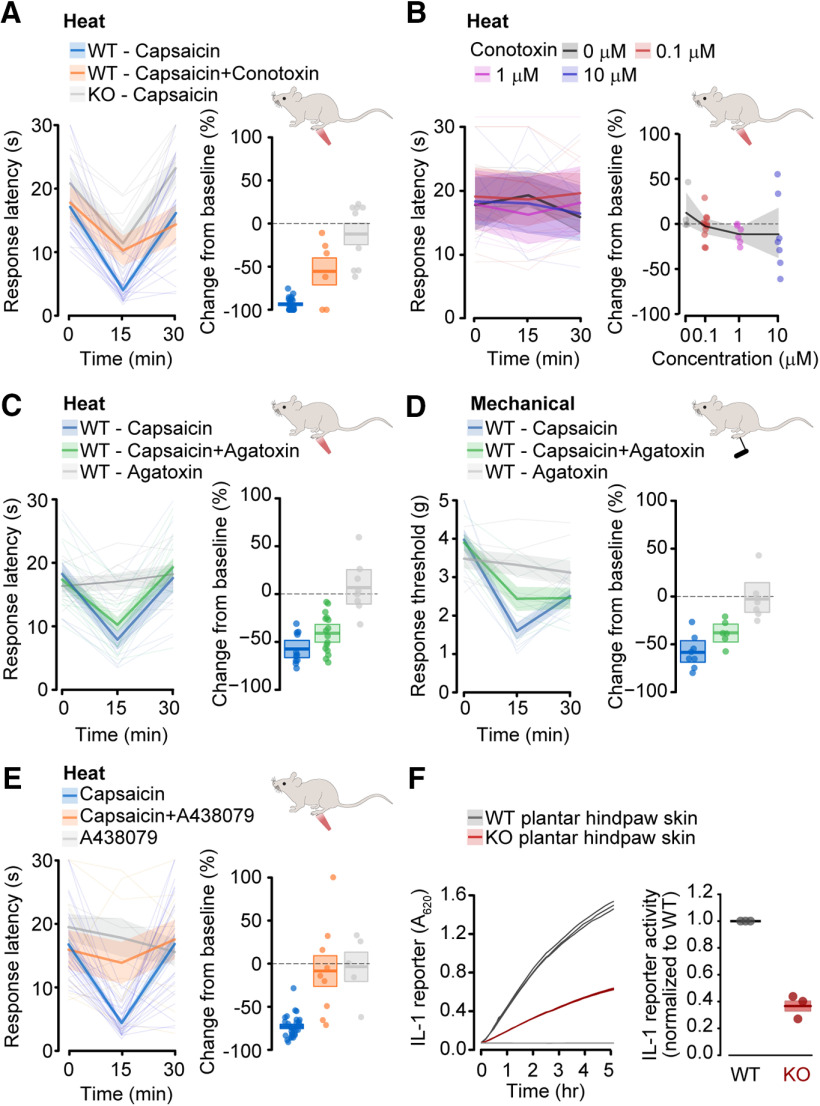
Capsaicin-induced heat hypersensitivity requires peripheral Ca_V_2.2 channels. ***A***, Heat sensitivity of plantar hindpaw of WT and KO mice measured before (0 min) and 15 and 30 min after injection of 0.1% w/v capsaicin in WT (blue, *n* = 23, same data as in Figure 4*A*), 0.1% w/v capsaicin + 1 μm ω-conotoxin MVIIA in WT (orange, *n* = 6), and 0.1% w/v capsaicin in KO (gray, *n* = 9, same data as in Figure 4*A*). Left, Responses from individual mice (thin lines, dashed and solid), average data (thick lines), and SE (shaded areas) are shown for all conditions. Right, Heat responses represented as change from baseline relative to saline for individual mice (symbols), average (horizontal line), and SE (shaded areas). Mean and SE percent change were for 0.1% w/v capsaicin + 1 μm ω-conotoxin MVIIA in WT: −56.2 ± 16.0%, χ^2^ = 4.185, *p* = 0.041 (WT capsaicin vs WT capsaicin + ω-conotoxin MVIIA) by Kruskal–Wallis test. ***B***, Heat sensitivity of plantar hindpaw of WT mice measured before (0 min) and 15 and 30 min after injection of the following different concentrations of ω-conotoxin MVIIA (0, 0.1, 1, 10 μm). Experiments were conducted by two experimenters under blinded conditions. For all plots, symbols are values measured from individual animals, average values (solid lines), and SE (shaded areas); *F* and *p* values calculated by univariate ANOVA for main effect of dose were *F* = 0.414 and *p* = 0.835. ***C***, Heat sensitivity of plantar hindpaw of WT mice measured before (0 min) and 15 and 30 min after injection of 0.1% w/v capsaicin alone (blue, *n* = 11), 1 μm ω-Agatoxin IVA alone (gray*, n* = 8), and 1 μm ω-Agatoxin IVA with 0.1% w/v capsaicin (green*, n* = 16). Experiments were conducted by one experimenter under blinded conditions. Left, Responses from individual mice (thin lines), average data (thick lines), and SE (shaded areas) are shown for all conditions. Right, Heat responses represented as change from baseline for individual mice (open symbols), average (horizontal line), and SE (shaded areas). Mean and SE were for Capsaicin: −57.5 ± 4.8%; Capsaicin + Agatoxin: −41.0 ± 5.1%; Agatoxin: 6.9 ± 9.8%; *F* and *p* values calculated by univariate ANOVA and Tukey's HSD correction for multiple comparisons are for Capsaicin versus Agatoxin, *F* = 40.69, *p* = 0.0000006; Capsaicin versus Capsaicin + Agatoxin, *F* = 5.030, *p* = 0.130. ***D***, Mechanical sensitivity of plantar hindpaw of WT mice measured before (0 min) and 15 and 30 min after injection of 0.1% w/v capsaicin alone (blue*, n* = 8), 1 μm ω-Agatoxin IVA alone (gray*, n* = 7), and 1 μm ω-Agatoxin IVA with 0.1% w/v capsaicin (green*, n* = 6). Experiments were conducted by one experimenter under blinded conditions. Left, Responses from individual mice (thin lines), average data (thick lines), and SE (shaded areas) are shown for all conditions. Right, Mechanical responses represented as change from baseline for individual mice (open symbols), average (horizontal line), and SE (shaded areas). Mean and SE were for Capsaicin: −58.4 ± 6.1%; Capsaicin + Agatoxin: −37.9 ± 5.2%; Agatoxin: −2.7 ± 8.4%; *F* and *p* values calculated by univariate ANOVA and Tukey's HSD correction for multiple comparisons are for Capsaicin versus Agatoxin *F* = 29.76, *p* = 0.00,003; Capsaicin versus Capsaicin + Agatoxin, *F* = 5.997, *p* = 0.118. ***E***, Heat sensitivity of plantar hindpaw of WT mice measured before (0 min) and 15 and 30 min after injection of 0.1% w/v capsaicin (blue, *n* = 28, including 23 from Figure 5*A*), 0.1% w/v capsaicin + 6 mm A438079 (orange*, n* = 9), or 6 mm A438079 (gray, *n* = 5). Left, Responses from individual mice (thin lines), average data (thick lines), and SE (shaded areas). Right, Heat responses represented as change from baseline at 15 min for individual mice (symbols), average (horizontal line), and SE (shaded areas). Mean and SE for 0.1% w/v capsaicin were −73.5 ± 2.5, for 0.1% w/v capsaicin + A438079 were −8.63 ± 18.3%, and for A438079 were −4.15 ± 17.0% change from baseline. Significance relative to capsaicin alone assessed by pairwise Wilcoxon test with Holm correction for multiple comparisons, was for capsaicin + A438079, *p* = 0.0001, and for A438079, *p* = 0.0002. ***F***, IL-1 release from plantar hindpaw skin from WT and KO mice was quantified using a cell-based reporter assay. Left, Data from individual experiment with technical triplicates showing reduced IL-1 reporter activity using media conditioned with skin from a KO mouse. Right, Summary showing results from three independent biological samples, normalized to WT reporter activity by experiment. Significance assessed by *t* test was *t* = −13.1, *p* = 0.006.

**Figure 6.**
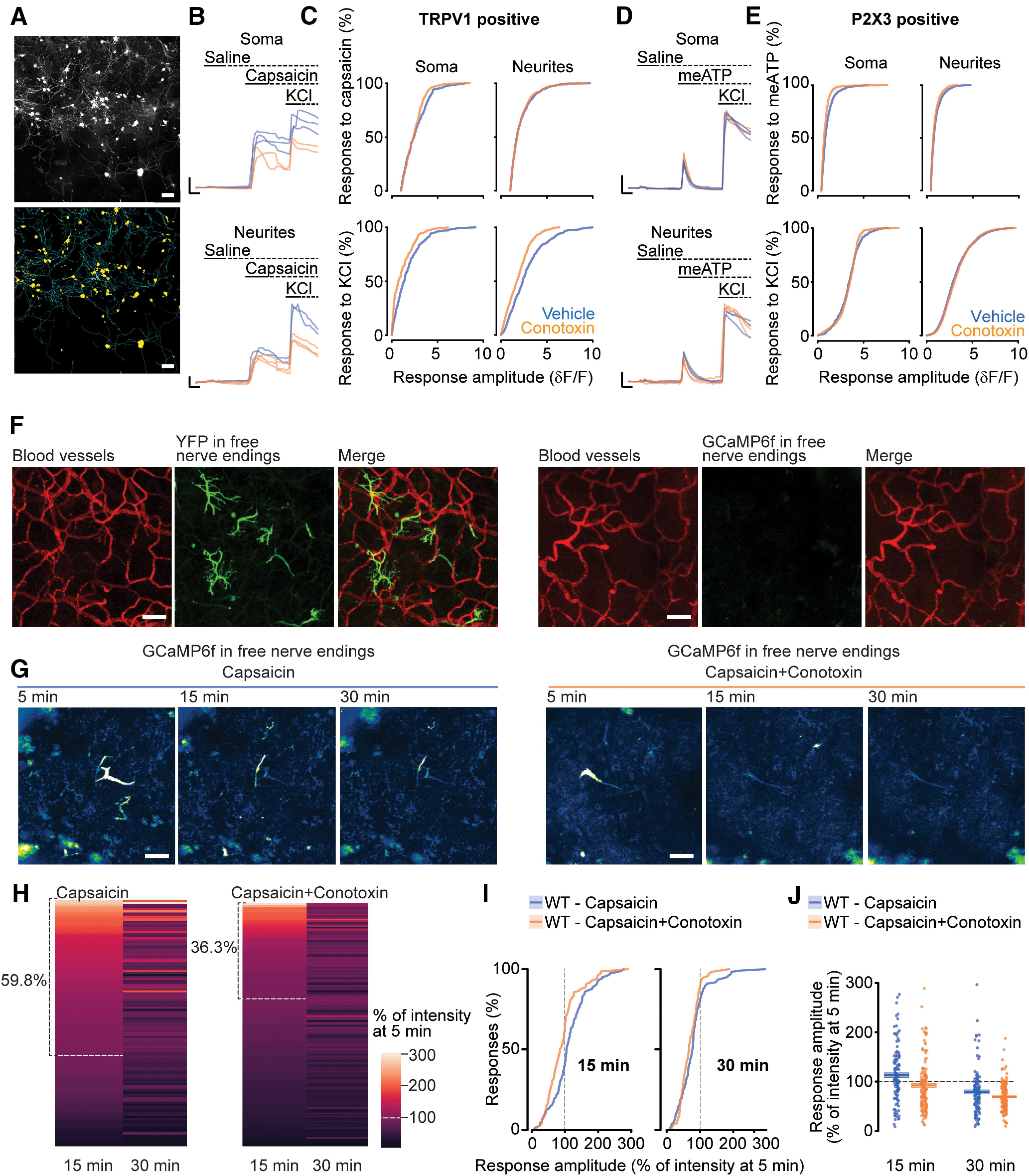
Ca_V_2.2 channels contribute to capsaicin-induced global calcium signals in Trpv1-lineage nociceptors nerve endings *in vivo*, which are not apparent in immature cultures. ***A***, Dissociated DRG stained with Fluo4 after 4 d in culture (top) along with processed image masks (bottom) identifying soma (yellow) and neurites (cyan). Scale bars: 100 μm. ***B***, Calcium signals measured in soma (top) and neurites (bottom) of capsaicin-sensitive neurons. Responses to consecutive applications of saline, 0.25 μm capsaicin, and 100 mm KCl, without (blue) or with (orange) 3 μm ω-conotoxin-MVIIA. Scale bars: 1 δF/F and 5 s. ***C***, Cumulative distributions of calcium response amplitudes measured in soma (left) and neurites (right) in response to 0.25 μm capsaicin (top) and 100 mm KCl (bottom) without (blue) or with (orange) 3 μm ω-CgTx MVIIA. Only neurons that respond to capsaicin are included in this population. *D* and *p* values calculated by Kolmogorov–Smirnov were for capsaicin responses in soma, *D* = 0.131, *p* = 0.038 (*n* = 285 vehicle and *n* = 194 conotoxin-treated from 3 mice); neurites, *D* = 0.030, *p* = 0.901 (*n* = 657 vehicle-treated and *n* = 810 conotoxin-treated from 3 mice); and for KCl in TRPV1-positive soma, *D* = 0.206, *p* = 0.0001 (*n* = 285 vehicle and *n* = 194 conotoxin-treated from 3 mice); and neurites, *D* = 0.186, *p* = 3 × 10^−11^ (*n* = 657 vehicle-treated and *n* = 810 conotoxin-treated from 3 mice). ***D***, Calcium signals measured in soma (top) and neurites (bottom) of αβ-meATP-sensitive neurons. Responses to consecutive applications of saline, 500 μm αβ-meATP, and 100 mm KCl, without (blue) or with (orange) 3 μm ω-conotoxin-MVIIA. Scale bars: 1 δF/F and 5 s. ***E***, Cumulative distributions of calcium response amplitudes measured in somas (left) and neurites (right) in response to 500 μm αβ-meATP (top) and 100 mm KCl (bottom) without (blue) or with (orange) 3 μm ω-conotoxin-MVIIA. Only neurons that respond to αβ-meATP are included in this population. *D* and *p* values calculated by Kolmogorov–Smirnov are for αβ-meATP responses in somas, *D* = 0.145, *p* = 0.00,005; neurites, *D* = 0.073, *p* = 0.0006; and for KCl responses in P2X3-positive soma *D* = 0.075, *p* = 0.116 (*n* = 673 vehicle treated, 405 conotoxin treated from 3 mice); and neurites, *D* = 0.045, *p* = 0.090 (*n* = 1639 vehicle treated, 819 conotoxin treated from 3 mice). ***F***, Reconstructed Z-stack images of two-photon imaging of plantar hindpaw *in vivo*. Left, *Trpv1^Chr2EYFP^*^+/−^ mice. Three panels: Dextran Texas-Red (red, in blood vessels), Trpv1-nociceptors (green), and merged. *Right*, *Trpv1^GCaMP6f^*^+/−^ mice. Three images, Dextran Texas-Red (red, in blood vessels), GCaMP6f signal (green), and merged. Scale bar, 50 μm. Note there is no background GCaMP6f signal under control naive conditions. Pulse source laser simultaneously tuned to 920 nm for GCaMP6f, and 1040 nm for dextran Texas-Red. ***G*** Two-photon functional imaging showing reconstructed Z-stack images for Trpv1-lineage nociceptor nerve endings (green) in *Trpv1^GCaMP6f^*^+/−^ mice after 5, 15, and 30 min intraplantar capsaicin (left), or coinjection with ω-conotoxin-MVIIA (right). Scale bar, 30 μm. ***H***, Heat map showing GCaMP6f calcium signals for each individual nerve at 15 and 30 min time points in response to capsaicin alone (left) or capsaicin with ω-conotoxin-MVIIA (right). Data are normalized to maximum capsaicin-induced response at 5 min. The normalization to the 5 min time point was necessary to compare across nerves and across animals, and the 5 min time point was the earliest signal we could acquire following motion distortion from the intraplantar capsaicin injection. We only analyzed nerves that showed a positive response to capsaicin. ***I***, Cumulative distribution of response amplitude for GCaMP6f signal in free nerve endings in skin after 15 (left) and 30 min (right) intraplantar capsaicin (blue), or coinjected with ω-conotoxin-MVIIA (orange). Data normalized to max GCaMP6f signal at 5 min. Significance, assessed by Kolmogorov–Smirnov test, for 15 min responses, *D* = 0.253, *p* = 0.0004, and 30 min responses, *D* = 0.141, *p* = 0.1427 (*n* = 122 nerves, 3 mice capsaicin alone; *n* = 146 nerves, 3 mice capsaicin with ω-conotoxin-MVIIA). ***J***, Response amplitude of GCaMP6f signal at 15 (left) and 30 min (right) following intraplantar capsaicin alone (blue) or coinjected with ω-conotoxin-MVIIA (orange). Responses are represented as percent change from the 5 min maximum response to capsaicin for each nerve analyzed (closed symbols) together with the average (horizontal line), and SE (shaded areas) for 268 nerves from six mice. Significance, assessed by repeated measures ANOVA with Tukey's HSD *post hoc* correction for multiple comparisons, for 15 min response, *t* = −3.246, *p* = 0.0011, and 30 min response, *t* = −1.565, *p* = 0.2712 (*n* = 122 nerves, 3 mice capsaicin alone; *n* = 146 nerves, 3 mice capsaicin with ω-conotoxin-MVIIA). Data sampling and analyses were conducted under blinded conditions.

Hypotheses were tested using R (version 3.5.0) and RStudio (version 1.1.463; Figs. 2*C*, 4, 5, 6) or IBM SPSS statistics software (version 24; Figs. 2*F*, 3). Before hypothesis testing, normality was assessed using the Shapiro–Wilks test, and homogeneity of group variances was assessed using Levene's test. For data that met normality and homogeneity of group variances assumptions (Fig. 2*C*,*F*, 3*B*,*C*, 4*A*,*B*,*D*, 5*B*,*E*,*F*, 6*J*), values were compared by ANOVA with Tukey's HSD correction for multiple comparisons where appropriate. If either assumption was not met (Figs. 4*E*, 5*A*,*C*), groups were compared by pairwise Wilcoxon tests with Holm's correction for multiple comparisons. Probability distributions (see Fig. 6*E,I,J*) were compared by Kolmogorov–Smirnov. Statistical testing results, including *p* values, are provided in the figure legends.

## Results

### Ca_V_2.2 channels contribute to heat and mechanical sensitivity

Ca_V_2.1 and Ca_V_2.2 channels support transmitter release from presynaptic sensory termini to postsynaptic dorsal horn neurons in the spinal cord, and they support thermal and mechanical hyperalgesia/allodynia that develop in chronic pain with reported, but poorly understood, differences in efficacy across models (Matthews and Dickenson, 2001; McGivern and McDonough, 2004; Zamponi et al., 2015). Ca_V_2.2 channel activity supports the induction and maintenance of chronic pain in nerve injury and inflammatory models (Chaplan et al., 1994; Yamamoto and Sakashita, 1998; Matthews and Dickenson, 2001), whereas, Ca_V_2.1 channels are more frequently invoked in the maintenance of secondary mechanical hypersensitivity associated with inflammation (Diaz and Dickenson, 1997; Sluka, 1997). Furthermore, the contribution of Ca_V_2.2 channels and their isoforms to mechanical and heat sensitivity remain incompletely defined (Hatakeyama et al., 2001; Kim et al., 2001; Saegusa et al., 2001; Bell et al., 2004; Jiang et al., 2013).

We used Ca_V_2.2 KO and exon-substitution knock-in mouse strains (Fig. 1; Andrade et al., 2010; Jiang et al., 2013) to quantify the overall role of Ca_V_2.2 channels and e37a splice isoforms to mechanical and heat sensitivity. We measured behavioral responses to conventional and optogenetic stimuli in WT, KO, and e37b-only strains (Fig. 2), and used immunoblotting and RNA *in situ* hybridization in DRGs to confirm the absence of Ca_V_2.2 protein in Ca_V_2.2 KO mice (Fig. 2, KO); the presence of Cacna1b RNA in DRG neurons (Fig. 2*B*, WT); and the presence of Cacna1b-e37a RNA in a subset of DRG neurons (Fig. 2*B*, WT).

Paw withdrawal latencies to heat were consistently longer (1.3-fold) in KO mice compared with WT controls (*p* = 0.0008; Fig. 2*C*). KO mice also had higher mechanical paw withdrawal thresholds (1.3-fold) compared with WT controls (*p* = 0.021; Fig. 2*C*). Our results support findings of some (Hatakeyama et al., 2001; Kim et al., 2001), but not all (Saegusa et al., 2001), previously reported studies using independently generated Ca_V_2.2 KO mouse strains.

These data underscore the importance of Ca_V_2.2 channels for heat and mechanical sensitivity but they also show that other Ca_V_ channels in addition to Ca_V_2.2 support behavioral responses to heat and mechanical stimulation. Previously, we observed similar reductions in heat and mechanical sensitivity in WT mice following pharmacological inhibition of Ca_V_2.2 channels by intrathecal ω-CgTx MVIIA (Jiang et al., 2013), suggesting that there is little or no compensatory increase in the expression of other Ca_V_ channels in the Ca_V_2.2 KO mouse strain used here. This conclusion is consistent with studies of other Ca_V_2.2 KO strains (Hatakeyama et al., 2001; Kim et al., 2001; Saegusa et al., 2001).

### Ca_V_2.2 channels contribute to LED-evoked behavioral responses

Trpv1-lineage axon fibers in the plantar hindpaw are primarily peptidergic nociceptors based on an overlap of Trpv1-lineage reporter (TdTomato) with anti-CGRP (Fig. 2*E*; Hsieh et al., 2012; Usoskin et al., 2015). We used LED stimulation of Trpv1-lineage ChR2-EYFP-expressing nerves in the plantar hindpaw to confirm the importance of Ca_V_2.2 channels in Trpv1-lineage nociceptors to behavioral and synaptic currents in spinal cord slices (Figs. 2*D–F*, 3). Brief exposure of the plantar hindpaw to blue LED light elicited robust and stereotyped nocifensive paw withdrawal responses in WT and KO mice that were light-intensity dependent (Fig. 2*F*). Compared with WT mice, KO mice were significantly less responsive to LED stimulation over a wide range of light intensities (WT, *n* = 17 mice; KO, *n* = 9 mice; *p* = 2 × 10^−6^, Fig. 2*F*). Behavioral differences in WT and KO mice were largest at submaximal LED intensities (Fig. 2*F*).

We compared LED-induced behavioral responses in exon-substituted e37b-only mice to WT (WT express e37a and e37b; Fig. 1) and conclude that e37b is able to functionally substitute for e37a. This conclusion is based on the similar behavioral responses evoked by heat, mechanical, and LED stimuli in a comparison of WT and e37b-only mice (heat, *p* = 0.624, Fig. 2*C*; mechanical, *p* = 0.228, Fig. 2*C*; LED, *p* = 0.997, Fig. 2*F*). These data support previous experiments using heat and mechanical stimuli (Andrade et al., 2010).

### Smaller postsynaptic currents in spinal cord dorsal horn neurons elicited by optical stimulation in Ca_V_2.2 KO mice compared with wild type

We used LED to activate Trpv1-lineage neuron afferent fibers in spinal cord dorsal horn acute slices from *WT/Trpv1*^Chr2EYFP+/−^ (WT), *Cacna1b*^−/−^*/Trpv1*^Chr2EYFP+/−^ (KO), and *Cacna1b*^e37b*-*e37b*+/+^/*Trpv1*^Chr2EYFP+/−^ (e37b-only) mice to compare postsynaptic currents. LED-evoked postsynaptic currents were recorded in lamina II neurons. Trpv1-lineage afferent fibers terminate in laminae I, II, and III, as demonstrated by the expression of ChR2-EYFP in areas both superficial to and deeper than IB4 binding (Fig. 3*A*). The total charge and amplitude of light-evoked postsynaptic currents (PSCs) were consistently smaller in neurons from KO mice compared with those in WT controls (PSC amplitude, *p* = 0.008; PSC total charge, *p* = 5.1 × 10^−5^; Fig. 3*B*). Light-evoked PSCs in KO neurons decayed more rapidly compared with WT as reflected by a more transient, and smaller, slow component of the light-evoked PSC (*p* = 0.009; Fig. 3*B*). By contrast, light-evoked PSCs in acute slices from e37b-only mice were not consistently different from WT with respect to amplitude, total charge, and decay time (e37b amplitude, *p* = 0.950; Fig. 3*B*). This parallels the behavioral sensitivity observed in e37b-only mice compared with WT (Fig. 2*F*).

Differences in the degree of attenuation of postsynaptic current amplitudes during repetitive stimulation is suggestive of presynaptic involvement (Manabe et al., 1993; Debanne et al., 1996; Dobrunz and Stevens, 1997), and the paired-pulse stimulation protocol is used frequently to assess presynaptic contributions (Zucker and Regehr, 2002; Jackman and Regehr, 2017).

We applied 10 light pulses at 1 Hz to acute spinal cord slices from WT, KO, and e37b-only mice (Fig. 3*C*). In all three genotypes, postsynaptic currents followed 1 Hz stimulation perfectly with no synaptic failures (Fig. 3*C*), but the degree of depression at KO synapses was consistently reduced compared with WT and e37b-only synaptic currents (KO, 86% of the initial EPSC amplitude compared with 51% and 54% in WT and e37b-only, respectively; WT vs e37b, *p* = 0.940; WT vs KO, *p* = 0.001; Fig. 3*C*). These results are consistent with reduced neurotransmitter release probability at nociceptor synapses lacking Ca_V_2.2 channels compared with WT (Scheuber et al., 2004; Nanou and Catterall, 2018; Held et al., 2020).

A predominately presynaptic role of Ca_V_2.2 channels in the spinal cord is well accepted (Diaz and Dickenson, 1997; Heinke et al., 2004; Motin and Adams, 2008; Jayamanne et al., 2013). But our data show (1) a reduction in synaptic transmission in our Ca_V_2.2 KO model consistent with its presynaptic localization (decrease in amplified and increase in paired pulse facilitation) and (2) no measurable difference in the efficacy of synaptic transmission in our Ca_V_2.2 e37b-e37b mouse model (no change in amplitude or paired-pulse facilitation). Ca_V_2.2 channels thus regulate behavioral sensitivity and contribute to transmission of information about heat and mechanical stimuli. At higher stimulus intensities, the recruitment of additional nerve fibers likely compensates for overall reduced release probability observed in KO mice at a naturalistic stimulation frequency.

We next asked if Ca_V_2.2 channels contribute to the classic hypersensitivity response induced by intradermal capsaicin, which is a widely used, robust model of transient hypersensitivity to heat and mechanical stimuli (Gilchrist et al., 1996; Caterina et al., 2000). Ca_V_2.2 channels, and Ca_V_2.2-e37a channel isoforms in particular, have been preferentially implicated in the development of several pain states including hyperalgesia (Diaz and Dickenson, 1997; Lee et al., 2003; Altier et al., 2007; Williams et al., 2008; Lee, 2013; Patel et al., 2015, 2018).

### Ca_V_2.2 channels are critical for capsaicin-induced heat hypersensitivity

Capsaicin activates neuronal transient receptor potential vanilloid 1 (TRPV1) channels to induce heat hyperalgesia and mechanical hypersensitivity. We used intraplantar injections of capsaicin (0.1% w/v), and at 15 and 30 min after injection, measured heat and mechanical hypersensitivity in WT, KO, and e37b-only mice (Fig. 4*A*). In all three mouse strains, we observed robust, spontaneous paw withdrawal responses immediately following capsaicin injection, which were gone within 5 min. This indicated successful injection of active capsaicin. Before capsaicin injection, paw withdrawal latencies in KO animals were longer (heat) and occurred at higher thresholds (mechanical) compared with WT mice, consistent with data shown in Figure 2*C*. We define heat hypersensitivity as a consistent reduction relative to baseline in the latency to paw withdrawal from a thermal stimulus (see above, Materials and Methods) and mechanical hypersensitivity as a reduced response threshold relative to baseline to a continually increasing mechanical stimulus from innocuous to noxious. Three experimenters using blinded protocols contributed to these data, and intraplantar capsaicin elicited heat hypersensitivity in all WT mice tested (*n* = 23 mice), whereas saline injection in WT mice had no effect (*n* = six mice).

We compared the effects of capsaicin and saline within each genotype to control for genotype-dependent effects of intraplantar injection itself and observed a striking difference in the effect of capsaicin relative to saline between WT and KO mice (*p* = 0.000004; Fig. 4*A*). WT mice consistently showed thermal hypersensitivity following intraplantar capsaicin relative to saline, whereas the effects of intraplantar saline and capsaicin in KO mice were indistinguishable. By contrast, the magnitude of capsaicin-induced mechanical hypersensitivity was not distinguishable between WT and KO animals (WT mice, *p* = 0.999; Fig. 4*B*). We conclude that Ca_V_2.2 channels contribute to capsaicin-induced transient heat hypersensitivity, but not to transient mechanical hypersensitivity, at least under the conditions of our experiments (Fig. 4*B*).

We next assessed capsaicin-mediated heat and mechanical hypersensitivity in e37b-only mice and found reduced heat hypersensitivity (e37b, *p* = 0.021; Fig. 4*A*) but not mechanical hypersensitivity (e37b, *p* = 0.862; Fig. 4*B*) relative to WT.

To control for the unlikely possibility that the global absence of Ca_V_2.2 (KO) or restricting e37 splicing options decreased capsaicin responsiveness, we compared capsaicin-induced calcium responses in DRG neurons isolated from WT, KO, and e37b-only mice (Fig. 4*C–E*) using an automated calcium imaging approach (DuBreuil et al., 2021). The percentage of cells responding to capsaicin (KO, *p* = 0.928; e37b, *p* = 0.995; Fig. 4*D*) were not different across mouse strains, and the amplitude of capsaicin-induced calcium responses of KO cells relative to WT (KO, *p* = 0.228; Fig. 4*E*) were not distinguishable, although there was a small but consistent shift toward larger amplitude responses in e37b-only cells relative to WT (e37b, *p* = 0.000005; Fig. 4*E*).

Our findings point to a new role for Ca_V_2.2 channels in capsaicin-induced heat hypersensitivity via TRPV1-expressing nociceptors. Our data also suggest that Ca_V_2.2-e37a channels may be necessary for the maximum heat hypersensitivity phenotype in response to dermal capsaicin. Ca_V_2.2 channels are completely absent in the Ca_V_2.2 KO mouse model at central and peripheral sites; therefore, we next used pharmacological inhibition of Ca_V_2.2 channels in WT mice to test whether local activation of Ca_V_2.2 channels in skin is essential for capsaicin-induced hypersensitivity.

### Peripheral Ca_V_2.2 channels are critical for capsaicin-induced heat hypersensitivity

We used intraplantar ω-CgTx MVIIA to inhibit peripheral Ca_V_2.2 channels and ω-Aga IVA to inhibit peripheral Ca_V_2.1 channels and measured the impact on capsaicin-induced heat and mechanical hypersensitivity (Fig. 5). Capsaicin injection induced immediate, short-term (up to 5 min) behavioral responses, including paw shaking, independent of intraplantar ω-CgTx MVIIA or ω-Aga IVA. Neither toxin alone affected behavioral responses to heat and mechanical stimuli (Fig. 5). We measured heat and mechanical hypersensitivity at 15 min and 30 min following capsaicin injection as the change from baseline in latency to paw withdrawal from a thermal stimulus and mechanical force required to elicit paw withdrawal (see above, Materials and Methods).

Heat sensitivity measured at 15 min was reduced by ω-CgTx MVIIA coinjected with capsaicin, relative to capsaicin alone (*p* = 0.041; Fig. 5*A*). These experiments provide strong evidence that local Ca_V_2.2 channel activation in skin is required for the development of capsaicin-induced heat hypersensitivity. Intrathecal ω-CgTx MVIIA attenuates basal nociception (Jiang et al., 2013; Wang et al., 2000); therefore, to control for the unlikely possibility that intraplantar ω-CgTx MVIIA reaches the spinal cord, we assessed behavioral responses in WT mice to heat in the presence of increasing concentrations of ω-CgTx MVIIA in the absence of capsaicin 15–30 min after injection (Fig. 5*B*). Intraplantar ω-CgTx MVIIA (0.1, 1, and 10 μm) did not impair behavioral responses to heat (*p* = 0.835, Fig. 5*B*), which is evidence that intraplantar ω-CgTx MVIIA does not reach the spinal cord, at least during the time course for these behavioral measurements.

DRG neurons including nociceptors and mechanoreceptors express multiple voltage-gated calcium channel genes, and both Ca_V_2.2 and Ca_V_2.1 channels trigger vesicular release of transmitters (Heinke et al., 2004; Zheng et al., 2019). To assess the specificity of Ca_V_2.2 channel involvement in capsaicin-induced heat hypersensitivity, we used the highly selective Ca_V_2.1 channel inhibitor ω-Agatoxin IVA. Intraplantar ω-AgaIVA (1 μm) had no effect on baseline heat-induced paw withdrawal responses and no effect on capsaicin-induced heat hypersensitivity (*p* = 0.130; Fig. 5*C*). Capsaicin-induced mechanical hypersensitivity was somewhat reduced on average by ω-AgaIVA (1 μm), although this effect was not consistent (*p* = 0.118; Fig. 5*D*). These data provide evidence that local activation of peripheral Ca_V_2.2, but not Ca_V_2.1 channels in skin are required for capsaicin-induced heat hypersensitivity.

Peripheral Ca_V_2.2 channels activated by capsaicin-induced membrane depolarization may trigger transmitter release from nociceptor nerve endings in the skin. Several inflammatory factors are released from nociceptor free nerve endings and contribute to neurogenic inflammation (Baraldi et al., 2004; Pankratov et al., 2006; Burnstock, 2007; Woolf and Ma, 2007; Teixeira et al., 2010; Chiu et al., 2012; Teixeira et al., 2014; Burnstock, 2016; Chai et al., 2017). Among these, ATP has been shown to act through P2X7 receptor signaling in inflammatory hyperalgesia (Teixeira et al., 2010; 2014).

We used the P2X7 antagonist A438079 to inhibit local ATP signaling in skin (Jarvis et al., 2001; Chessell et al., 2005; McGaraughty et al., 2007). Intraplantar A438079 (6 mm) attenuated capsaicin-induced heat hypersensitivity (*p* = 0.0001; Fig. 5*E*), consistent with ATP release downstream of TRPV1 channel activation. The involvement of P2X7 receptors in capsaicin-evoked hyperalgesia implicates non-neuronal cells, and P2X7 receptor activation triggers the release of the prominent inflammatory mediator IL-1 (Giuliani et al., 2017). This raises the possibility that Ca_V_2.2 channel activation might trigger downstream pathways that induce IL-1 release in skin. We compared the spontaneous release of IL-1 from isolated skin samples in WT and KO mice. We cultured plantar hindpaw skin from WT and KO mice for 72 h, collected conditioned media, and quantified IL-1 levels using a cell-based reporter assay. IL-1 levels from skin of KO mice were 40% of that from WT (*p* = 0.006, Fig. 5*F*). Our data suggest that P2X7 receptor activation is involved in capsaicin-mediated heat hypersensitivity and that spontaneous release of IL-1 from skin depends on the presence of Ca_V_2.2 channels.

### Ca_V_2.2 channels contribute to capsaicin-evoked intracellular calcium in peripheral axons of Trpv1-lineage nociceptors *in vivo* but not in immature neurites in culture

Our model predicts that capsaicin induces calcium influx through Ca_V_2.2 channels in TRPV1-nociceptors, presumably via TRPV1 receptor-mediated depolarization. To provide evidence for Ca_V_2.2-dependent calcium signals in TRPV1 nociceptors, we first measured intracellular calcium in soma and neurites of mouse primary DRG neurons grown for 4 d in culture (Fig. 6*A–E*). TRPV1-nociceptors were identified based on their responsiveness to capsaicin, and calcium signals were acquired in response to sequential capsaicin (250 nm) and high K^+^ (100 mm) stimulation, in the absence and presence of ω-CgTx MVIIA (3 μm; Fig. 6*B*). Treatment with ω-CgTx MVIIA reduced the amplitude of calcium signals elicited by high K^+^ in soma (*p* = 0.001) and neurites (*p* = 3*10^−11^) of TRPV1-nociceptors, but ω-CgTx MVIIA did not affect capsaicin-evoked calcium responses in neurites, although it did have a small but consistent effect on a subset of larger amplitude signals in soma (soma, *p* = 0.038, neurites, *p* = 0.901; Fig. 6*C*). For comparison, we also measured intracellular calcium in soma and neurites of nonpeptidergic P2X3-expressing nociceptors identified by sensitivity to αβ-methylene ATP (meATP; 500 μm; Shiers et al., 2020; Fig. 6*D*). In contrast to TRPV1-nociceptors, ω-CgTx MVIIA had much smaller effects on high K^+^-induced calcium responses in P2X3 neurons in neurites (*p* = 0.090) but small effects on larger amplitude intracellular calcium evoked by high K^+^ or meATP in soma (*p* = 0.116; *p* = 0.00,005), and in neurites across a slightly broader range of amplitudes (*p* = 0.006, Fig. 6*E*; North, 2016). These data confirm that TRPV1 nociceptors in culture express Ca_V_2.2 channels in both soma and neurites (Fig. 6*B*,*C*) and that TRPV1 receptor activation by capsaicin elicits a robust intracellular calcium response throughout the neuron, but TRPV1 activation by capsaicin did not elicit detectable calcium signals through Ca_V_2.2 channels in neurites in these cultures. Neurites in culture lack cell–cell interactions and other molecules that may regulate the precise composition or proximity of signaling molecules in axon termini in skin found *in vivo*.

We therefore established conditions to image intracellular calcium in Trpv1-lineage nociceptor axons innervating the plantar hindpaw *in vivo*. Using two-photon imaging of *Trpv1*^GCaMP6f+/−^ mice, along with vascular labeling by intravenous Texas Red dextran, we measured intracellular calcium signals in response to capsaicin in Trpv1-lineage nociceptor nerve endings of *Trpv1*^GCaMP6f+/−^ mice using the coimaged blood vessels as a reference both during image acquisition and postacquisition motion correction. By this approach, we visualized Trpv1-lineage nociceptor nerve endings reliably within 200 µm from epithelial skin cells, in the same focal planes as the blood vessel network (Fig. 6*F*).

We assessed intracellular calcium in Trpv1-lineage nociceptor axons elicited by intraplantar injection of capsaicin (0.1% w/v) alone or capsaicin with ω-CgTx MVIIA (1 μm). We analyzed >120 nerves for each condition and only unmasked the experimental conditions postanalyses. Capsaicin injection caused motion distortion that we could not correct at early time points, but within 5 min reliable image acquisition was possible (Fig. 6*G*,*H*). We therefore analyzed image stacks spanning the 200 µm immediately adjacent to the epidermis at 5, 15, and 30 min after injection (Fig. 6*G*). Basal levels of calcium-bound GCaMP6f were undetectable (Fig. 6*F*). All analyses were performed on axonal endings that responded within 30 min of capsaicin application (*n* = 120 fibers from three mice), and calcium response amplitudes were expressed as normalized values relative to the 5 min postinjection time point. Heat maps, cumulative plots, and scatter plots show that at 15 min postinjection, response amplitudes of ω-CgTx MVIIA-treated axon endings were decreased overall relative to the untreated fibers (*p* = 0.0004), whereas 30 min capsaicin response amplitudes were not consistently different on average between capsaicin and capsaicin plus ω-CgTx MVIIA (*p* = 0.1427; Fig. 6*I*). Capsaicin-induced intracellular calcium kinetics parallel behavioral responses to capsaicin. In the absence of ω-CgTx MVIIA, the average capsaicin-induced intracellular calcium response peaked at 15 min (Fig. 6*J*), whereas, in the presence of ω-CgTx MVIIA, the average intracellular calcium levels were reduced relative to the 5 min time point (Fig. 6*J*). These data show that Ca_V_2.2 channels in Trpv1-lineage nociceptors contribute to the intracellular calcium signal induced by capsaicin in nerve endings innervating the plantar hindpaw *in vivo*.

Our data reveal an underappreciated, essential, and selective role for Ca_V_2.2 channels in TRPV1-nociceptor peripheral axons in capsaicin-induced heat but not in mechanical hypersensitivity. TRPV1-nociceptor-specific Ca_V_2.2 channel splice isoforms containing e37a may have a unique role in capsaicin-induced heat hyperalgesia that is not fully supported by Ca_V_2.2 e37b splice isoforms.

## Discussion

Ca_V_2.2 channels are important targets of analgesic drugs and neuromodulators that regulate calcium entry at presynaptic terminals. Our data show that Ca_V_2.2 channel activity in TRPV1 nociceptor nerve endings in skin is critical for capsaicin-induced transient hyperalgesia to heat but not for mechanical hypersensitivity (Figs. 4, 5). Baseline responses were unaffected by intraplantar inhibition of Ca_V_2.2 channels (Fig. 5), pointing to a selective role for peripheral Ca_V_2.2 channels in controlling hypersensitivity to heat. As best we can tell, only one other group has evidence that peripheral Ca_V_2.2 channel activation is important for development of hypersensitivity. In a peripheral nerve injury model, daily intradermal inhibition of Ca_V_2.2 but not Ca_V_2.1 was reported to reduce mechanical hypersensitivity (White and Cousins, 1998). More recently, peripheral application of a dual Ca_V_2.2 and Na_V_1.8 inhibitor (CNCB-2) was shown to attenuate heat and mechanical hypersensitivity in postoperative and inflammatory pain models, although this study did not assess the role of Ca_V_2.2 directly (Lee et al., 2019). Aside from these studies, the field has essentially focused on the role of Ca_V_2.2 channels at central sites while overlooking the role of Ca_V_2.2 channels at peripheral nerve endings.

Our findings elucidate a key step involved in the capsaicin-induced inflammatory response in skin; establish a unique role for Ca_V_2.2 channels in capsaicin-induced heat, but not mechanical hypersensitivity; and demonstrate that local inhibition of Ca_V_2.2 channels can occlude certain forms of heat hypersensitivity independent of the role of Ca_V_2.2 channels in the transmission from peripheral to central sites (Hatakeyama et al., 2001; Kim et al., 2001; Saegusa et al., 2001). Our findings do not imply that central mechanisms are not in play; they must be. But we do show that interrupting the Ca_V_2.2-dependent calcium signal at peripheral sites can mitigate at least transient, and potentially more persistent, forms of heat hypersensitivity.

### Peripheral Ca_V_2.2 channels in skin required for heat hypersensitivity

Capsaicin-induced heat and mechanical hypersensitivity is a robust model of transient neurogenic inflammation. However, little or no attention has been paid to understanding how capsaicin-induced depolarization of TRPV1 nociceptors triggers an inflammatory response (Sweitzer et al., 2004). Our studies support a model in which Ca_V_2.2 channels in peripheral nerve termini are activated by capsaicin-induced depolarization, and we suggest that the calcium that enters nerve endings through Ca_V_2.2 channels is a critical early step in the development of hypersensitivity. Capsaicin can also trigger action potentials in DRG neurons (Blair and Bean, 2002; Castiglioni et al., 2006), which could potentially back propagate and contribute to calcium entry (Gossard et al., 1999; Gafurov et al., 2020), although it is not known if such signals could reach peripheral nerve termini innervating skin.

Exocytosis of peptidergic and ATP-containing vesicles is widely acknowledged to follow nociceptor activation (Louis et al., 1989; Chiu et al., 2012; Gouin et al., 2017), and our study suggests that Ca_V_2.2 channels are the critical link between these two steps in neurogenic heat hyperalgesia (Huang and Neher, 1996; Meng et al., 2007; Jung et al., 2014). In culture and *in vivo* calcium imaging showed that Ca_V_2.2 channels contribute to depolarization-mediated intracellular calcium in TRPV1 nociceptors, but we only found evidence of a link between TRPV1 receptor activation and Ca_V_2.2 channel activation *in vivo*. It is possible that neurites in culture lack cell–cell interactions and other molecules that may regulate the precise composition or proximity of signaling molecules in axon termini in skin found *in vivo*.

### Ca_V_2.2 channels in nerve endings in skin contribute global calcium signals

The contribution of Ca_V_2.2 calcium to the global intracellular calcium signal in nociceptors is relatively small because global calcium is derived from many sources including multiple calcium-permeable plasma membrane ion channels (TRPV1, TRPA1, Ca_V_1.2, Ca_V_1.3, Ca_V_2.1, Ca_V_2.2, and Ca_V_3.2) and calcium release from intracellular stores triggered by calcium (Lipscombe et al., 1988; Holliday et al., 1991; Llano et al., 1994; Usoskin et al., 2015; Renthal et al., 2020). Nonetheless, we found consistent evidence that Ca_V_2.2 channels contribute to the capsaicin-induced calcium signals in nerve endings in skin *in vivo*.

Ca_V_2.2 channels couple membrane depolarization to exocytosis of a range of neurotransmitter-containing vesicles including peptides (Waterman, 1997; Sher et al., 1998; Catterall, 1999; Pankratov et al., 2006; Kamp et al., 2012; Zamponi et al., 2015; Chai et al., 2017). Our data implicate Ca_V_2.2 channels in TRPV1 nociceptor nerve endings in skin in early events, likely transmitter release, that trigger capsaicin-induced heat hypersensitivity (Fig. 5; Cook and McCleskey, 2002; Burnstock, 2007; Jung et al., 2014; Burnstock, 2016), and it is possible that coincident calcium signals, through TRPV1 and Ca_V_2.2 channels, may be necessary to initiate this signaling cascade.

Capsaicin-induced heat hypersensitivity was eliminated by local P2X7 receptor block, and, as P2X7 receptors are located on non-neuronal cells (Kaczmarek-Hajek et al., 2018), our data implicate non-neuronal cells in the development of transient heat hypersensitivity induced by intraplantar capsaicin. For example, we show that spontaneous release of interleukin-1 is attenuated in skin from Ca_V_2.2 KO mice relative to wild-type control. Other inflammatory signals, including neuropeptides and cytokines, likely act in synergy with or downstream of ATP (Chiu et al., 2012), and Ca_V_2.2 channels may also contribute to release of these factors from microglia (Saegusa et al., 2001), sympathetic neurons (Lipscombe et al., 1989; Ren et al., 2005), or TRPV1 nociceptor nerve endings by lysosomal exocytosis or bulk release through dying cell pores (Cook and McCleskey, 2002; Burnstock, 2007; Jung et al., 2014).

### Ca_V_2.2 channels in nerve endings in skin specifically regulate heat hypersensitivity

**Figure 7.**
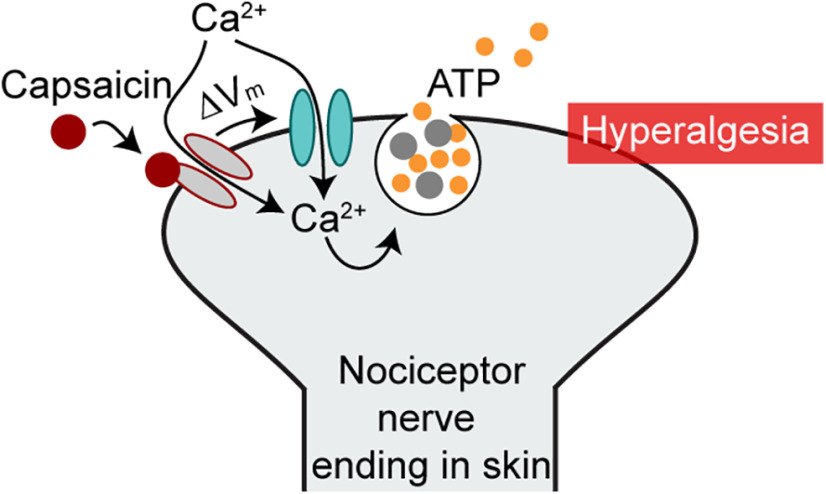
Proposed mechanism for Ca_V_2.2 channel involvement in capsaicin-induced heat hypersensitivity. Capsaicin binds TRPV1 channels on Trpv1-nociceptor nerve endings in skin, the nociceptor membrane depolarizes, Ca_V_2.2 channels open, calcium enters nociceptors through Ca_V_2.2 channels and through Trpv1 channels. Calcium entry through Ca_V_2.2 channels triggers ATP release from secretory vesicles, which acts via second order cells to trigger transient heat hypersensitivity.

Local block of Ca_V_2.2 channels in skin by ω-CgTx-MVIIA specifically attenuated capsaicin-induced heat hypersensitivity in mice while leaving mechanical hypersensitivity and basal nociception intact. This is consistent with the divergent signaling pathways that support heat and mechanical hypersensitivity (Fuchs et al., 2001; Liu and Ma, 2011; Ebbinghaus et al., 2012; Usoskin et al., 2015; Li et al., 2016; Ghitani et al., 2017). Peripheral inhibition of Ca_V_2.2 by ω-CgTx-MVIIA has been reported previously to attenuate mechanical hypersensitivity in a peripheral nerve injury model of chronic pain (White and Cousins, 1998), suggesting that in addition to a role in transient heat hypersensitivity, peripheral Ca_V_2.2 channels may also contribute to a positive feedback cycle of ongoing local release of pro-inflammatory mediators and nociceptor depolarization in chronic inflammation (Fig. 7).

### Peripheral inhibition of Ca_V_2.2 channels for analgesia

Ca_V_2.2 channels are important targets for the development of non-opioid analgesics, and although it has limited use, ω-CgTx-MVIIA relieves pain in patients with otherwise intractable chronic pain (McGivern, 2007; Zeilhofer et al., 2012; Lee, 2013; Zamponi et al., 2015; Yekkirala et al., 2017). Local application of ion channel blockers of Ca_V_2.2 (shown here) and a dual inhibitor of Ca_V_2.2, Na_V_1.7 and Na_V_1.8 (Lee et al., 2019) may have clinical utility against certain inflammatory responses by interrupting local reinforcing signals that contribute to neuronal sensitization. A peripheral site of action also circumvents the debilitating side effects that accompany spinal level administration of ω-CgTx-MVIIA (Miljanich, 2004).

### Peripheral Ca_V_2.2 channel slice isoforms

Ca_V_2.2-e37a channel splice isoforms, which are expressed in Trpv1-lineage nociceptors (Bell et al., 2004; López Soto and Lipscombe, 2020) are necessary for the complete expression of capsaicin-induced heat hypersensitivity (Fig. 4). By contrast, Ca_V_2.2-e37a and Ca_V_2.2-e37b channels appear to be fully interchangeable with respect to their function in supporting acute nociception (Figs. 2, 3; Andrade et al., 2010). Compared with Ca_V_2.2-e37b, Ca_V_2.2-e37a channels are trafficked more efficiently to the cell surface, and they are more susceptible to Gαi/o-dependent inhibition including by µ-opioid receptors (Raingo et al., 2007; Andrade et al., 2010; Marangoudakis et al., 2012; Jiang et al., 2013; Macabuag and Dolphin, 2015; Gandini et al., 2019; Lopez Soto et al., 2019). We speculate that Ca_V_2.2-e37a isoforms are trafficked with greater efficiency, compared with Ca_V_2.2-e37b isoforms, to peripheral axon endings in skin, accounting for their specialized role in capsaicin-induced heat hypersensitivity. Alternatively, Ca_V_2.2-e37a isoforms may colocalize preferentially with TRPV1 receptors. Isoform-selective inhibitors specifically targeting Ca_V_2.2-e37a channels might be expected to preferentially act on nociceptor Ca_V_2.2 channels at peripheral sites that mediate heat hypersensitivity, with fewer off-target effects and without affecting acute nociception (Bell et al., 2004; Altier et al., 2007; Andrade et al., 2010; Jiang et al., 2013).

Much progress has been made in identifying and inhibiting the downstream actions of inflammatory mediators that are released in response to chemical irritants, tissue injury, and in certain diseases. Our studies show that Ca_V_2.2 channels are critical for the action, and likely release, of early inflammatory mediators including ATP implicated in several animal models of the hypersensitivity to stimuli that follows transient and prolonged forms of neurogenic inflammation. Heat hypersensitivity develops in sensory nerves following capsaicin exposure, and this process depends on Ca_V_2.2 channel activation, and this has important implications for optimal strategies to reduce or prevent different types of pain pathologies.
